# Neural pathways mediating cross education of motor function

**DOI:** 10.3389/fnhum.2013.00397

**Published:** 2013-07-29

**Authors:** Kathy L. Ruddy, Richard G. Carson

**Affiliations:** ^1^School of Psychology, Queen's University BelfastBelfast, UK; ^2^Trinity College Institute of Neuroscience and School of Psychology, Trinity College DublinDublin, Ireland

**Keywords:** interlimb, bilateral, transfer, motor learning, interhemispheric

## Abstract

Cross education is the process whereby training of one limb gives rise to enhancements in the performance of the opposite, untrained limb. Despite interest in this phenomenon having been sustained for more than a century, a comprehensive explanation of the mediating neural mechanisms remains elusive. With new evidence emerging that cross education may have therapeutic utility, the need to provide a principled evidential basis upon which to design interventions becomes ever more pressing. Generally, mechanistic accounts of cross education align with one of two explanatory frameworks. Models of the “cross activation” variety encapsulate the observation that unilateral execution of a movement task gives rise to bilateral increases in corticospinal excitability. The related conjecture is that such distributed activity, when present during unilateral practice, leads to simultaneous adaptations in neural circuits that project to the muscles of the untrained limb, thus facilitating subsequent performance of the task. Alternatively, “bilateral access” models entail that motor engrams formed during unilateral practice, may subsequently be utilized bilaterally—that is, by the neural circuitry that constitutes the control centers for movements of *both* limbs. At present there is a paucity of direct evidence that allows the corresponding neural processes to be delineated, or their relative contributions in different task contexts to be ascertained. In the current review we seek to synthesize and assimilate the fragmentary information that is available, including consideration of knowledge that has emerged as a result of technological advances in structural and functional brain imaging. An emphasis upon task dependency is maintained throughout, the conviction being that the neural mechanisms that mediate cross education may only be understood in this context.

## Introduction

### General context

The capacity for activity of one limb to influence the subsequent performance of its opposite counterpart has been documented for more than a century. As early as 1894, Scripture and colleagues employed a simple manometer to demonstrate that unilateral strength training gives rise to enhanced performance of the same task by the untrained opposite limb. This effect—for which the term “cross education” was coined, has been reproduced in a plethora of research investigations, encompassing both the transfer of strength and motor skill (Laszlo et al., 1970; Parlow and Kinsbourne, 1989; Imamizu and Shimojo, 1995). Despite longstanding interest in the phenomenon, there is, however, little consensus concerning the mediating neural mechanisms.

Why is this knowledge deficit of more general significance? As a case in point, a significant risk associated with the fractures that arise from falls by older adults, is that the loss of specific muscle strength or general capacity resulting from limb immobilization will leave the person below the level of capability necessary to perform everyday tasks, and thus maintain independent living. Even in younger persons with extensive functional reserves, 3 weeks of immobilization leads to declines of strength in the order of 50% of initial capacity (Hortobagyi et al., 2000). If, however, the opposite limb is trained during the period of immobilization, the loss of functional capacity is attenuated (Farthing et al., 2009; Magnus et al., 2010; Pearce et al., 2012). Given this therapeutic potential, there is an obvious need to provide a principled basis upon which to design interventions and tailor these appropriately to address individual requirements.

### Scope of the review

While originally cross education was deemed to encompass the transfer of muscle strength following a period of unilateral resistance training, and the transfer of skill following unilateral skill training (Scripture et al., 1894), the majority of contemporary empirical studies have treated strength transfer and skill transfer as separate entities (Farthing, 2009). The conviction that the two facets of cross-education are intimately related underpins the present review. Specifically, the transfer of strength or vigor following a period of unilateral resistance or ballistic training, and the transfer of skill following a period of unilateral skill training appear to be mediated by shared mechanisms. That which is at issue is the precise nature of these mechanisms, and the degree to which their respective contributions vary in accordance with specific task demands.

**Figure 1:**
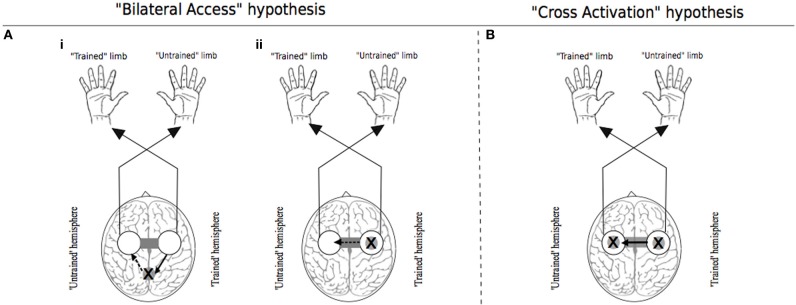
**Traditional theoretical models of cross education**. In each instance the “X” represents the putative locus of training related adaptations. White circles indicate lateralized motor networks in their entirety. In Panel **(A)**, solid arrows represent processes that occur during unilateral training. Dashed arrows represent processes that are specific to the subsequent transfer phase during which movements are generated by the untrained limb. **(Ai)** illustrates the hypothesis that engrams elaborated during unilateral motor training are established in brain centers that are accessible to the motor networks of both the trained and untrained limbs. **(Aii)** exemplifies the “callosal access” hypothesis, whereby training related adaptations are lateralized to motor networks projecting to the trained limb, and are accessible also to motor networks projecting to the untrained limb via callosal transfer. Panel **(B)** represents the “cross activation” hypothesis that during unilateral training, activation of the homologous motor network gives rise to bilateral adaptations that facilitate subsequent performance by the untrained limb. Adapted from Lee et al. (2010).

With regard to the extant literature, two principal theoretical models can be delineated (Figure 1). The first is derived from observations that the execution of many unilateral tasks is associated with increased excitability of both contralateral and ipsilateral cortical motor areas. The principal tenet of the “cross-activation” model is that bilateral cortical activity generated during unilateral training drives concurrent neural adaptations in both cerebral hemispheres. Accordingly, unilateral training induces task specific changes in the configuration of cortical motor networks that normally control the muscles of the opposite (quiescent) limb (Hellebrandt, 1951). Since the magnitude of cross-activation is contingent on the intensity of the unilateral contraction (Perez and Cohen, 2008), the degree of transfer is predicted to scale with the level of neural drive required to perform the training task. The “bilateral access” model (Laszlo et al., 1970; Taylor and Heilman, 1980; Imamizu and Shimojo, 1995) holds that motor “engrams” elaborated during unilateral training are not specific to the control of trained limb. Rather they are encoded in a more abstract fashion, at a locus that is also accessible for the control of the opposite untrained limb (Anguera et al., 2007). In this scheme, the degree of transfer is predicted to vary with the complexity of the training task (Farthing, 2009).

The primary goal of the present review is to elucidate the neural pathways that mediate cross education of motor function. In principle, one might also aim to assess the degree to which the structural and functional properties of the brain networks thus identified are commensurate with the respective theoretical models. To do so, however, it is first necessary to identify grounds upon which the models can be considered distinct. By Reductio ad Absurdum, transfer via cross-activation would be restricted to homologues of the effectors engaged during training (Davis, 1898). Conversely, transfer via bilateral access would be effector independent (Latash, 1999). We argue that the mechanisms that mediate the cross education of motor function are most profitably considered in relation to such factors as the characteristics of the training task (e.g., Sosnik, 2010). While the terms “Cross Activation” (section) and “Bilateral Access” (section) are retained for presentation purposes, we consider the empirical findings not only in relation to the eponymous models, but also ask whether it is possible to achieve a deeper appreciation of the mediating neural pathways through means other than their contrast. By and large our analysis is focused upon upper limb movements.

## Cross activation

### Theoretical framework and empirical evidence

In proposing that “some grooving of the neuronal pattern thought to be essential to motor learning must take place on the ipsilateral side, while the main stream of descending impulses flows to the contralateral limb,” Hellebrandt (1951) was making appeal to two lines of evidence. The first was derived from observations that in a large cohort of school children, the developmental increase in strength, rate, and precision of movement exhibited by the preferred right hand was not markedly larger than that of the non-preferred hand—in spite of the much greater use presumed of the former (Bryan, 1892). The original author was led to conclude “the effects of use on the right side have been shared by the corresponding joints on the left side” (page 201). The second was the report by Welch (1898) that when a maximum intensity grip was generated by one hand, there was activation of the muscles of the opposite hand, and indeed muscles in other parts of the body. This is the phenomenon of motor irradiation. When considered specifically in relation to effectors or muscles (i.e., of the opposite limb) that are homologues of those engaged in focal contractions, such terms as associated movements (Todor and Lazarus, 1986), mirror movements (Mayston et al., 1999) and contralateral irradiation (Cernacek, 1961) have been applied. Hellebrandt was perhaps the first to draw an explicit link between the presence of this phenomenon and the cross education (or bilateral transfer) of motor function. It was, however, presaged by Davis (1898) more than a century ago. While the potential origins of cross education may appear obvious in circumstances in which training movements of one limb give rise to associated movements of the other limb, cross activation may be latent and yet still have functional consequences in relation to the transfer of strength or skill.

As a result of technological advances in recent years, it has become possible to probe the nature of such latent interactions. Bilateral variations in the excitability of corticospinal projections during movements that are by intention unilateral, have been demonstrated using transcranial magnetic stimulation (TMS). Motor evoked potentials (MEPs) induced by TMS are increased in amplitude by isometric contractions of the homologous muscles in the opposite forearm (Hortobagyi et al., 2003). The amount of potentiation, or “crossed-facilitation,” is positively correlated with the amount of force that is generated by the contractions of the opposite limb (Perez and Cohen, 2008). In the case of rhythmic movements, MEPs evoked in the quiescent muscles of a static limb vary in accordance with the phase of motion of the opposite (moving) limb (Carson et al., 1999, 2004). The MEP is maximally potentiated during the phase in which the homologous muscle in the opposite (moving) limb is maximally activated. Since corresponding changes in response amplitude are not obtained when potentials are evoked by stimulating the corticospinal pathway at the level of the cervico-medullary junction (Hortobagyi et al., 2003; Carson et al., 2004), it has been concluded that the phenomenon of crossed-facilitation has inter-hemispheric interactions between cortical motor areas as its primary physiological basis.

While it is evident that these interactions find expression via corticospinal output from M1, it cannot be assumed that direct interactions between the primary motor cortices represent the source of crossed facilitation. In monkey, mirror movements are abolished by the temporary inactivation (through injection of muscimol) of M1 ipsilateral to the actively moving limb, whereas they are largely preserved, or indeed enhanced, in circumstances in which the opposite M1 (i.e., contralateral to the moving limb) is injected (Tsuboi et al., 2010). This pattern of outcomes suggests that crossed facilitation arises from common drive to both primary motor cortices from other centers in the motor network. In a related vein, it has been noted (Cisek et al., 2003) that in non-human primates, there is a strong correlation between the directional tuning of cells in dorsal premotor cortex (PMd) during reaching movements made by the ipsilateral and contralateral limb, whereas for primary motor cortex the degree of association is markedly lower. During rhythmic contractions of a finger muscle performed by humans, connectivity from the contralateral (to movement) PMd to ipsilateral M1– as assessed by paired-pulse TMS techniques, is modulated by variations in contraction frequency (Uehara et al., 2013).

Human neuroimaging provides complementary evidence. Although activity in ipsilateral M1 is elevated during unilateral movements (Singh et al., 1998b; Dai et al., 2001; Kobayashi et al., 2003; van Duinen et al., 2008), greater increases are typically registered in areas anterior, lateral and ventral to the primary motor cortex, in a region on the precentral gyrus that most likely corresponds to premotor cortex (Kawashima et al., 1997; Singh et al., 1998a; Cramer et al., 1999; Kobayashi et al., 2003; Stanćák et al., 2003; Koeneke et al., 2004; Hanakawa et al., 2005; Verstynen et al., 2005; Horenstein et al., 2009; Verstynen and Ivry, 2011; Diedrichsen et al., 2013). The firing rate of neurons in this region, when recorded directly in primate models, relates to movement parameters such as acceleration and velocity (Kubota and Hamada, 1978), extent of movement, direction and amplitude (Fu et al., 1993; Kurata, 1993). Similarly in humans, activity in ipsilateral premotor cortex is modulated by task parameters that dictate the level of neural drive that must be directed to the muscles of the active limb. Elevations in cerebral blood flow related to movement velocity have been reported for ipsilateral premotor cortex, anterior cerebellum, superior parietal lobule and basal ganglia (Turner et al., 1998), and corresponding to movement frequency in ipsilateral premotor cortex and cerebellum (Jenkins et al., 1997). During repetitive key tapping movements, as the level of force necessary to depress the key is increased from 5 to 60% maximal voluntary contraction (MVC), there is a pronounced increase in regional cerebral blood flow (rCBF) in primary motor cortex ipsilateral to the active hand (Dettmers et al., 1996). Similarly, BOLD signal intensity registered in ipsilateral M1 scales with the applied level of force (Dai et al., 2001; van Duinen et al., 2008). In this regard, it is notable that when comparisons are made within individual studies (e.g., Walters, 1955), or across studies (Zhou, 2000), the degree of cross education appears to be contingent upon the level of voluntary drive generated during training.

In seeking to establish whether activity generated during unilateral training drives concurrent adaptations in both cerebral hemispheres that are sufficient to increase the functional capacity of the untrained limb, it is thus necessary to assess the totality of neural pathways and mechanisms that may play a causal role. Recognizing that in all natural tasks control is achieved through the balanced modulation of inhibitory and facilitatory processes, it is also important to consider whether specific variations in this balance may arise through training, be subject to chronic adaptation over varying time courses, and exert a functional effect upon movements of the opposite limb.

### The concept of crossed surround inhibition

Studies in cat indicate there are facilitatory connections with the homotopic area of the opposite motor cortex that are surrounded by a more extensive zone in which inhibitory responses to transcallosal stimulation may be obtained (Asanuma and Okuda, 1962). Single unit studies further reveal that there is wider dispersion of inter-spike intervals in the peripheral (inhibitory) zone, suggesting a greater number of interceding synaptic relays (Kogan and Kuraev, 1976). This is consistent with the conjecture that callosal neurons are glutamatergic (Werhahn et al., 1999) and facilitatory to their immediate targets (Houzel and Milleret, 1999). Thus, the extent to which the output of primary motor cortex invokes crossed inhibition is contingent on neural interactions that converge upon circuits local to the opposite hemisphere (Bianki and Shrammapril, 1985; Berlucchi et al., 1990; Daskalakis et al., 2002; Carson, 2005).

**Figure 2:**
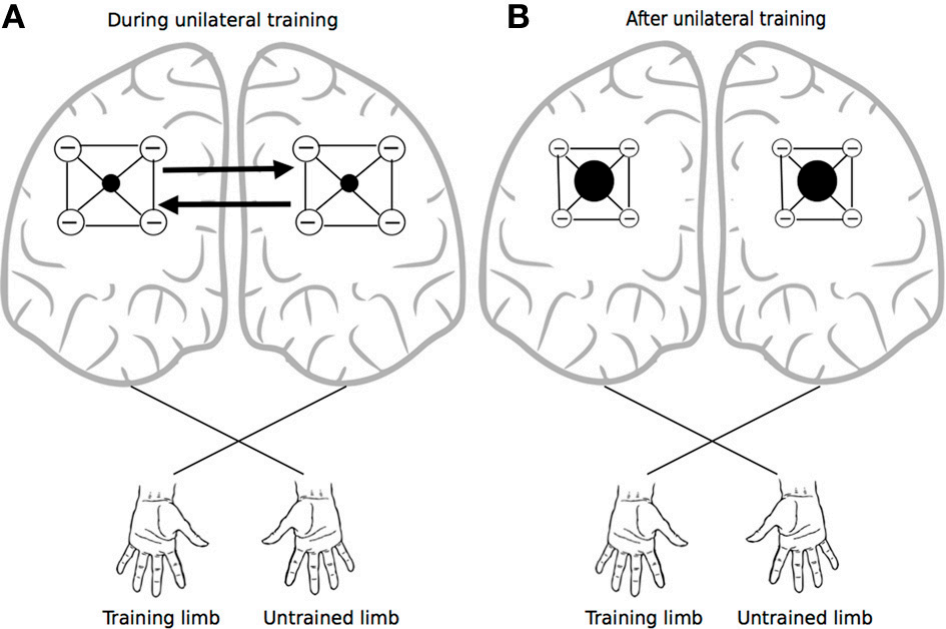
**Crossed reciprocal surround inhibition**. The black filled circles represent excitatory nodes within intracortical circuits (i.e., in both hemispheres) that project to the focal muscles engaged by the training task. These are surrounded by a more extensive inhibitory zone. Following Bianki (e.g., Bianki and Makarova, 1980; Bianki and Shrammapril, 1985), Panel **(A)** represents interactions that occur during unilateral training. Panel **(B)** illustrates the altered state that results from unilateral training: the excitatory foci are enlarged, and surround inhibition is reduced.

It has been proposed that this mode of organization provides a means of focusing activity in thalamocortical relays—via surround inhibition (Beck and Hallett, 2011), an effect that is attenuated markedly by callosal section (Bianki, 1981). Importantly in the present context, the narrowing of the excitatory focus that occurs through this means is thought to be reciprocal in nature (Bianki and Makarova, 1980). Increases in surround inhibition in one hemisphere give rise to a reverse (i.e., symmetrical and selective) influence on the contralateral hemisphere (Bianki and Shrammapril, 1985). If the modulation of intracortical inhibition by means of callosal projections (Figure 2) is indeed reciprocal (e.g., Pal, 2005), the changes that occur in the organization of the homologous representation of the muscles engaged in training can be conceived of as being functional and adaptive, rather than simply incidental. From a broader phylogenetic perspective, it would appear likely that the mechanisms underlying cross education have bestowed fitness beyond the range of circumstances that are the subject of contemporary interest.

It has long been supposed that inhibition in general (Welch, 1898), and surround inhibition in particular (e.g., Denny-Brown, 1967), plays a crucial role in the selective recruitment of the focal muscles engaged in a task, and (i.e., with training) the disengagement of muscles with actions that might otherwise interfere with the desired movement outcome (Carson, 2006). Nonetheless, it remains to be determined whether surround inhibition arises from interactions local to cortex, or is mediated by inputs from other nodes of the motor network (Beck and Hallett, 2011). Duque et al. (2008) have previously raised the possibility that changes in surround inhibition may be one factor mediating the bilateral neuroplastic adaptation that results from unimanual training. The empirical literature concerning changes in surround inhibition that may occur as a consequence of training is, however, somewhat sparse. It is not even clear that surround inhibition can be revealed in all individuals using current TMS-based measures (e.g., Kang et al., 2012). Repetitive movements requiring the selective engagement of a single effector lead to diminution of MEPs and increased intracortical inhibition (inferred using paired-pulse TMS techniques) in other hand muscles (Liepert et al., 1998; Bütefisch et al., 2005). Conversely, following tasks that require synchronized movements of two fingers the opposite effect is obtained (Kang et al., 2012). We are not aware of any studies that have directly examined variations in surround inhibition in the context of cross education.

### Experimental indices of interhemispheric inhibition and facilitation

The possibility has been raised elsewhere that unilateral practice exerts its chronic effects on the functional capacity of the opposite limb through modification of the focal excitatory relationship between the primary motor cortices and/or the characteristics of the inhibition engendered by intracortical circuits (Hortobagyi, 2005). Task- and practice-dependent variations in these processes, and their balance, are however, challenging to resolve using the tools currently available in human electrophysiology.

It is well-documented that if an initial magnetic (conditioning) stimulus is applied to one primary motor cortex shortly (6–15 ms) before a second (test) stimulus is directed to the other M1, the magnitude of the response to the test stimulus is reduced (Ferbert et al., 1992). If however, the test response is evoked by transcranial electrical stimulation (TES), magnetic conditioning stimuli applied to the opposite hemisphere have no such effects (Ferbert et al., 1992; Hanajima et al., 2001), thus corroborating the assumption that inhibitory effects of M1 inter-hemispheric projections are mediated by local interneurons. While such inter-hemispheric inhibition (IHI) is more readily elicited in the laboratory, facilitation may also be obtained (Ugawa et al., 1993; Salerno and Georgesco, 1996; Hanajima et al., 2001; Baumer, 2006). The interval between the conditioning stimulus and the test stimulus is typically shorter than that required for IHI (Salerno and Georgesco, 1996), and the effect is most readily precipitated when the conditioning is either by TES or medially directed magnetic stimulation (Hanajima et al., 2001).

In seeking to use these techniques as a means of elucidating the neural basis of cross education, it is necessary to consider whether they are capable of discriminating changes in the excitatory balance between the primary motor cortices caused by unilateral training, from alterations in excitatory-inhibitory interactions within local interneuron circuits in the hemisphere ipsilateral to the training limb. It would also be advantageous to first demonstrate that they are capable of resolving the basis of acute variations in corticospinal excitability, such as those obtained *during* contractions of muscles in the ipsilateral limb. In this regard, tonic or pulsed isometric contractions are typically used as experimental paradigms (for a review see Perez, 2012).

In a condition in which the sizes of the conditioned and test MEP were matched across torque levels, Perez and Cohen (2008) reported that IHI measured in the resting flexor carpis radialis muscle (FCR) during isometric flexion of the opposite wrist was lower when torque was generated at 30% and 70% maximum voluntary contraction (MVC) than at 10% MVC (see also Chiou et al., 2013). In contrast, when the MEPs generated by the conditioning stimulus (CS) were not matched across conditions, an increase in IHI was obtained. This accords with the outcomes of other studies in which matching of the conditioning stimulus intensity was not performed (Ferbert et al., 1992; Vercauteren et al., 2008; Talelli et al., 2008a). Hinder et al. (2010b) reported a similar upwards scaling of IHI during pulsed applications of force (5% to 30% MVC).

A small number of studies concern changes in IHI arising as a result of short-term practice. Bologna et al. (2012) required that individuals maximize the initial acceleration of ballistic abduction movements of the (right—dominant) index finger, while attempting to maintain constant the level of activity (at 5–10% MVC) recorded from the homologous [first dorsal interosseus (FDI)] muscle of the opposite limb. Practice consisted of 100 repetitions of the movement at a rate of ~ 0.2 Hz. Prior to and following these movements IHI (adjusted CS and TS intensities) was assessed at rest using interstimulus intervals of 12 ms (prototypical short-latency) and 30 ms (long-latency). Although the practice-related improvements in performance were accompanied by an increase in the excitability of corticospinal projections from the contralateral M1 (i.e., to the training limb), there were no corresponding changes in IHI (i.e., from the “trained” to “non-trained” hemisphere). In contrast, in the context of a task that required modulation of precision pinch (index finger and thumb) grip to acquire a sequence of five force targets, improvements in the speed and accuracy of performance of the (non-dominant) left hand were observed following 180 training trials performed by the right hand (over 30 min). This positive transfer of learning was accompanied by a decrease in IHI (“trained” to “non-trained” hemisphere)—estimated using adjusted CS and TS intensities (Camus et al., 2009).

In one of the only studies conducted thus far in which potential variations in IHI have been assessed in the context of chronic training protocols, Hortobagyi et al. (2011) engaged volunteers to participate in 20 training sessions, conducted over 8 weeks, during which 1000 submaximal (80% MVC) applications of (abduction) force by the right index finger were undertaken. The maximum force applied by the trained finger was elevated by 49.9% as a consequence of the intervention, and the untrained finger exhibited an increase of 28.1%. Measures of IHI (“trained” to “non-trained” M1) were obtained at rest prior to the intervention and after every fifth session, using CS intensities that were fixed, and TS intensities that were adjusted (within and across sessions) for each participant. Similar estimates were also recorded at the beginning and end of these specific training sessions. It was reported that IHI decreased by 30.9% over the course of the entire intervention, and acutely by 8.9% on average during single sessions. In addition when the degree of cross education was correlated (across participants) with changes in IHI, the level of covariation was observed to increase over the course of the intervention.

The findings of Hortobagyi et al. (2011) provide a strong indication that the chronic effects of unilateral training upon movements of the opposite limb are mediated, at least in part, by processes manifested via TMS derived IHI (assessed at rest). This interpretation is not without some caveats. The conclusions that are drawn on the basis of the IHI technique can depend profoundly on the control of conditioning stimulus intensities. For example, in circumstances in which both the CS and TS are fixed, an increase in IHI is obtained with elevations in contraction (ipsilateral to TS) intensity (e.g., Ferbert et al., 1992; Perez and Cohen, 2008; Talelli et al., 2008b; Vercauteren et al., 2008). If however, the stimulation intensity is adjusted to maintain constant the amplitude of the conditioning MEP, experimentally elicited (short-latency) IHI is attenuated with increases in the level of contraction (Perez and Cohen, 2008; Chiou et al., 2013).

A more general challenge is thereby illustrated—that of relating measures of interhemispheric interaction obtained from conscious humans using non-invasive (e.g., magnetic) brain stimulation to those derived from reduced animal preparations. The ipsilateral silent period (iSP) provides another index of interhemispheric inhibition. It is obtained when TMS is delivered at high intensity to the M1 ipsilateral to contracting muscles (Wassermann et al., 1991). The spread of activation at these intensities appears to mask any excitatory effects, thus giving rise to net inhibition of the opposite motor cortex. As with IHI, the initial portion of the iSP appears to be mediated, at least in part, by the fibers of the corpus callosum (Meyer et al., 1995, 1998). Nonetheless, short-latency (e.g., 8 ms interval) IHI and iSP do not vary equivalently in response to a number of experimental manipulations (Chen et al., 2003; Giovannelli et al., 2009). The greater covariation observed for the iSP and long-latency (e.g., 40 ms interval) IHI suggests that these respective effects may be subserved by overlapping subpopulations of neurons (Chen et al., 2003).

If considered in relation to the variations that are manifested at different levels of isometric contraction, the area of the iSP (i.e., the degree of inhibition of EMG activity ipsilateral to the stimulation) is greater during both minimal (5% MVC) and maximum engagement of the homologous (FDI) muscle (i.e., opposite limb) than when it is quiescent (Giovannelli et al., 2009). These outcomes are thus consistent with those obtained for short-latency IHI, when fixed CS and TS intensities are employed, since both measures of inhibition scale with the intensity of the contraction performed by the homologous muscle. Notably in respect of Giovannelli et al. (2009), increases of iSP area were also obtained during (maximal) contraction of the opposite extensor indicis proprius (EIP), but not with contraction of more proximal upper limb muscles or lower limb muscles, suggesting that the effect is topographic but not entirely focal (see also Hinder et al., 2010b). To the best of our knowledge, the iSP technique has not yet been used to investigate interlimb transfer of training.

How is the ostensible elevation in inter-hemispheric inhibition (i.e., fixed CS intensity IHI; iSP) that occurs during the course of unilateral ballistic (e.g., Duque et al., 2007) and isometric contractions (Vercauteren et al., 2008), and rises with the intensity of contraction (e.g., Perez and Cohen, 2008; Giovannelli et al., 2009), to be reconciled with the decreases that are measured acutely (at rest) during the course of a unimanual training session (e.g., Camus et al., 2009; Hortobagyi et al., 2011) and chronically over multiple sessions (Hortobagyi et al., 2011), and which may be related to the cross education of motor function that is observed in such circumstances? One possibility is that *during* voluntary contractions, the excitability of transcallosal projections is modulated in parallel with that of corticospinal neurons (Avanzino et al., 2007). While this will give rise to increases in both inter-hemispheric facilitation and inhibition, it may simply be the case that the experimental techniques that are typically employed (e.g., IHI and iSP) do not provide an adequate representation of variations in the local balance between excitation and inhibition that occur in the ipsilateral M1 during voluntary movement. We are, for example, unaware of any attempts to apply paired-pulse TMS techniques to examine levels of inter-cortical facilitation in these contexts. Alternatively, the decreases in IHI that are registered when the CS intensity is adjusted (downwards) during unilateral contractions may be gauged more representative of processes that are implicated in cross education of function. It is also noteworthy that experimentally elicited (short-latency) IHI is abolished when forces greater than 50% MVC are generated by the muscle in which the test MEP is recorded (Chen et al., 2003). It seems likely that the decreases in IHI observed as a result of training—in contexts in which cross education is obtained, express alterations in the excitatory-inhibitory balance within interneuron circuits local to the hemisphere ipsilateral to the training limb, rather than changes in the characteristics of the projections between the primary motor cortices that are recruited at rest by magnetic stimulation. Such acute (i.e., within a single training session) and chronic (i.e., across multiple training sessions) alterations may arise in association with, for example, increases in surround inhibition induced during training by reciprocal interactions between the hemispheres (Bianki and Shrammapril, 1985). In summary, experimental indices of inter-hemispheric inhibition (and facilitation) provide only a partial indication of the relationship between the physiological processes that are operative during the execution of the training movements, and thus of the neural pathways that mediate their cumulative functional consequences.

### The nature and role of crossed facilitation

The evidence that voluntary contractions of one limb—at the intensities employed in training regimes, give rise to increases in the excitability of descending projections to the homologous muscles of the opposite limb is incontrovertible. Furthermore, many of the factors that modulate this crossed facilitation are also those that, when manipulated, alter the level of cross education that is brought about by unilateral training. Perhaps the strongest indication that the two phenomena are functionally related is provided by the recent report that crossed facilitation registered during background contractions (20% and 80% MVC) of the trained (homologous) muscle of the opposite limb, increased over the course of 20 training sessions. These changes were correlated highly with the level of cross education (Hortobagyi et al., 2011).

When repetitive movements are performed using a distal effector of one limb, the frequency with which clearly distinguishable EMG activity is registered in the homologous muscles of the opposite limb increases when the focal movements are subject to external resistance (Cernacek, 1961), or performed with greater effort (Hopf et al., 1974). Similarly, if the muscles of the active limb are progressively fatigued, there is a corresponding increase in the EMG activity recorded from the opposite limb (Arányi and Rösler, 2002). These data are consistent with the proposal that the extent of irradiation to the opposite limb is contingent upon the level of neural drive directed to the muscles engaged in the focal movement (Todor and Lazarus, 1986). In the absence of voluntary drive, when a limb is moved passively, functional neuroimaging techniques generally fail to reveal signal change in ipsilateral cortex, despite the registration of activity in the hemisphere contralateral to movement (Francis et al., 2009; Yu et al., 2011; Szameitat et al., 2012). There is also at least one proposal that, at low levels of force, unilateral contractions suppress ipsilateral motor cortical activity (Liepert et al., 2001). In this regard, it has been suggested that lower levels of crossed facilitation during low force tasks, particularly when these are bimanual, may serve to prevent interference between the limbs. Conversely, the presence of motor irradiation during high force movements is ostensibly advantageous in carrying heavy loads when bilateral cooperation is desirable (e.g., Liepert et al., 2001). This conjecture is, however, inconsistent with the widely noted bilateral force deficits expressed in circumstances in which maximal levels of motor output are demanded simultaneously (Ohtsuki, 1983; Archontides and Fazey, 1993).

It has long been recognized that cross facilitation effects persist beyond the period of training. The phenomenon, which was first dubbed the “aftercontraction effect” (Craske and Craske, 1986), may also be detected on the basis of changes in corticospinal output in response to TMS (e.g., Carson et al., 2008). In the context of tasks in which short-term unilateral practice engenders bilateral improvements in performance, sustained increases in the excitability of corticospinal projections to the muscles of the untrained limb (recorded at rest) have been reported in acute (Carroll et al., 2008; Lee et al., 2010; Hinder et al., 2011; Poh et al., 2013) and chronic (Koeneke et al., 2006) ballistic training protocols. Corresponding outcomes have been obtained during (acute) practice of precision grip force modulation (Liang et al., 2007). It cannot be assumed, however, that such changes are of adaptive functional significance, since they are obtained not only for homologues of the muscles engaged in the training task, but also for homologues of muscles that do not make a *direct* mechanical contribution to the action that is trained (Carroll et al., 2008). In addition, there is evidently no relationship across participants between the degree of cross education and increases in the excitability of corticospinal projections to the homologues of muscles engaged in training, when these are assessed at rest in the context of either acute (Carroll et al., 2008; Hinder et al., 2011) or chronic (Hortobagyi et al., 2011) training protocols. On these grounds, it would appear reasonable to consider whether the functional adaptations that underpin interlimb transfer of gains in performance either occur in areas upstream of the primary motor cortex, or via changes in the effectiveness of synaptic transmission through projections from these areas onto M1 targets.

It has been reported that short- interval intracortical inhibition (SICI) increases during isometric contractions of the ipsilateral limb (Perez and Cohen, 2008) and the modulation of precision pinch grip force (Camus et al., 2009), whereas corresponding effects have not been obtained for ballistic movements (Hinder et al., 2010a). The observation that the effect of IHI conditioning on SICI invoked within the M1 ipsilateral to contractions is stronger during efforts at 70% MVC than at rest, suggests either that at least some of the modulation of intracortical circuits mediating SICI occurs via direct input from the opposite hemisphere (Perez and Cohen, 2008) or that these circuits are interposed with interneurons (i.e., in the target hemisphere) that are engaged in the expression of IHI. Nonetheless, neither of these putative mechanisms provide a direct account of the influence of factors such as vision on levels of cross facilitation (Carson et al., 2005; Garry et al., 2005; Carson and Ruddy, 2012) in circumstances in which the descending output from the active M1 does not vary across conditions (see also Avanzino et al., 2007). Furthermore, measures of intracortical inhibition (SICI) and facilitation (ICF) do not change systematically within (McCombe Waller et al., 2008) or across training sessions, and thus these measures do not correlate with the induced levels of transfer (Hortobagyi et al., 2011). This lack of association suggests that the processes that mediate the expression of SICI and ICF (when assessed at rest) are incidental to those that underlie cross education. While it remains to be resolved whether a local recasting of the inhibitory-excitatory balance as characterized by variations in ICF or SICI, is promoted directly by variations in the state of transcallosal neurons projecting from the homologous “active” M1 during unilateral contractions, the alterations in intracortical excitability thus expressed do not appear functionally related to changes that are instrumental in relation to the interlimb transfer of gains in performance realized through repeated training.

As far as we are aware, only a single study has used a perturbation approach to gain insight in relation to the locus of adaptations that underlie cross education. As the results of this study are amenable to a number of alternative interpretations, it is worth considering in some detail. Lee et al. (2010) asked their participants to perform 300 ballistic movements with a view to maximizing acceleration of the right index finger. The peak acceleration of the trained finger increased by 93%, and that of the untrained (left) finger increased by 62%. When rTMS (15 min at 1 Hz) was applied subsequently to the right M1—contralateral to the untrained limb, the peak acceleration of the left index finger was attenuated by 15.5% (i.e., relative to the value obtained immediately following the cessation of training). In contrast, the performance of the trained right finger was unchanged. In the complementary condition, in which rTMS was administered to the (left) M1 contralateral to the training limb, the peak acceleration of the right index finger was attenuated by 13.1%. There was no corresponding diminution of performance for the left index finger. For the groups that performed active training movements, there was marked elevation of MEPs evoked in the target muscle of the trained and untrained limbs following the 300 movements (prior to rTMS), suggesting increases in the excitability of the output circuits from both primary motor cortices. As noted previously, such changes do not appear instrumentally related to levels of transfer (Carroll et al., 2008; Hinder et al., 2011). It seems unlikely therefore that the disruptive impact of the rTMS upon performance was realized through the M1 circuits that are recruited in generating a corticospinal volley in response to single pulse TMS. It has been highlighted recently that the state of the cortex at the time of stimulation (e.g., rTMS) both determines the overall neuronal response of the stimulated cortex, and shapes the responsiveness of distinct subpopulations of cortical neurons (Siebner et al., 2009). The functional consequences of rTMS on the output of M1 are therefore likely to be quite different if delivered at rest (or in control conditions in which no preceding movements are performed), or in circumstances in which the stimulated neurons have been preconditioned by movements of the contralateral or ipsilateral limb (as in the key experimental conditions of the Lee et al. study). Thus, it is possible that the performance decrements observed by Lee and colleagues following rTMS reflected disturbance of interneuronal networks other than those directly engaged in generating corticospinal output. Conceivably these networks include the intracortical circuitry that is engaged in IHI protocols, and which exhibits adaptation related in extent to the level of cross education (Camus et al., 2009; Hortobagyi et al., 2011). Alternatively, the effects of the rTMS on task performance may simply reflect attenuation of the net M1 response to synaptic input from other brain areas, rather than disruption of mechanisms acting within M1 that are specifically related to cross education.

The interpretation that the output circuits of the primary motor cortex ipsilateral to the training limb are the conduit rather than the wellspring of cross-limb transfer is likewise consistent with reports that unilateral strength training increased the capacity of the motor cortex to drive the homologous untrained muscles (Lee et al., 2009). These observations serve to illustrate the more general point that, at least with respect to resistance training and other “maximal output” paradigms, chronic adaptations are often only revealed in circumstances in which output circuits of the primary motor cortex receive synaptic drive (i.e., during voluntary contractions). This appears to be the case both for the untrained (Hortobagyi et al., 2011) and the trained limb (Griffin and Cafarelli, 2007; Carroll et al., 2009). Indeed, since it is by no means established that resistance training engenders adaptations in M1 output circuitry contralateral to the limb that is directly engaged (Carroll et al., 2002; Jensen et al., 2005), even in circumstances in which transmission via this area necessarily approaches maximum levels during training, it would appear counterintuitive if the crossed effects were to be mediated by this means.

### Sources of functional connectivity

It is necessary to consider whether there are other sources of bilateral functional connectivity within the motor network that have the potential to mediate cross activation, and provide a mechanism for cross education of function (Farthing et al., 2007). During unilateral movement, ipsilateral activation has been reported not only for M1 and premotor cortex, but also in regions including supplementary motor cortex (SMA) (Dai et al., 2001; Diedrichsen et al., 2013), primary sensory cortex (S1) (Dai et al., 2001; Kobayashi et al., 2003), cerebellum (Dai et al., 2001; van Duinen et al., 2008; Horenstein et al., 2009), parietal lobe (Dai et al., 2001; Hanakawa et al., 2005; van Duinen et al., 2008; Horenstein et al., 2009), and cingulate cortex (Dai et al., 2001).

**Figure 3:**
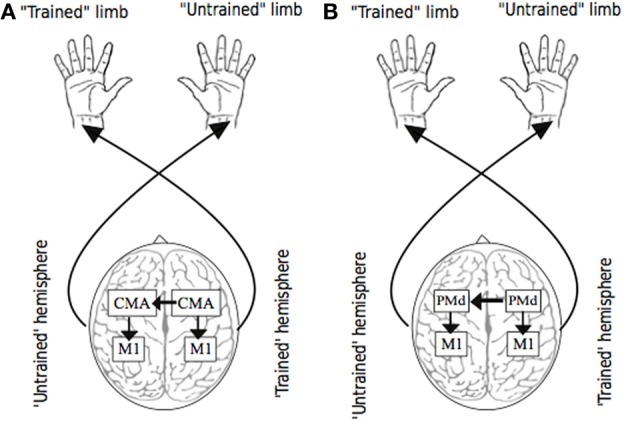
**Putative sources of transfer**. Panel **(A)** illustrates the possibility that as a consequence of (transcallosal) interactions between cingulate motor areas (CMA) during unilateral training, bilateral adaptations occur in circuits projecting from CMA, including those to targets within M1. Panel **(B)** illustrates the possibility that as a consequence of (transcallosal) interactions between dorsal premotor cortices (PMd) during unilateral training, bilateral adaptations occur in circuits projecting from PMd, including those to targets within M1.

For example, activity in the cingulate motor area (CMA)—which forms part of the anterior cingulate cortex and is thought to be a strategic entry point for limbic influence on the voluntary motor system, is closely associated with the amount of effort demanded by a motor task (Winterer et al., 2002). The observation that a high proportion of CMA neurons exhibit activity that is modulated when the ipsilateral hand is engaged (Kermadi et al., 2000), is consistent with the widespread finding that crossed facilitation is accentuated with increased effort or volition (Hopf et al., 1974). In primates, the cingulate motor area (CMA) is very densely connected with its homologue in the other hemisphere via fibers of the corpus callosum (Rouiller et al., 1994). In addition, functional connectivity between the caudal ACC and the primary and supplementary motor areas in humans is now clearly established (Koski and Paus, 2000). Thus, it is conceivable that the bilateral activity registered in elements of the motor network during unilateral movement, arises first in the cingulate cortex of the contralateral hemisphere, extends through callosal fibers to the ipsilateral CMA, and subsequently to other (ipsilateral) motor areas before influencing M1 output (Carson et al., 2005) (Figure 3). This conjecture is supported by the observation that in neurologically healthy human subjects, the activity registered in cingulate cortical areas during unimanual movements is correlated positively with the size of the posterior truncus of the corpus callosum (Stanćák et al., 2003). Taken together, these findings suggest that the level of input from regions such as anterior cingulate cortex that appear to act as neural mediators of the central command (e.g., Chefer et al., 1997) may also determine the bilateral distribution of activity across elements of the cortical motor network that arises during the effortful engagement of a single limb.

### The structural basis of cross activation

What are the possible grounds for evaluating the proposal that cross education of motor function is mediated by mechanisms acting via neural pathways projecting from areas upstream of the primary motor cortices? A necessary but not sufficient step is to assess bilateral structural connectivity with a view to delineating the routes by which such functional interactions might occur.

In non-human primates, the density of callosal connections exhibits a rostrocaudal gradient for the M1, SMA-proper, and pre-SMA (Liu et al., 2002), whereby the hand representation in primary motor cortex is relatively sparsely connected with its contralateral counterpart (Jenny, 1979; Rouiller et al., 1998). The pre-SMA, which is believed to be involved in early phases of motor preparation and planning, exhibits much denser callosal connectivity than the SMA-proper or primary motor cortex. The scope for direct inter-hemispheric interactions via callosal pathways thus decreases progressively along a functional gradient that culminates in those regions that have the most prominent role in generating motor output. In the context of bimanual movement, it has been proposed previously that this organization is consistent with the requirement that inter-hemispheric interference at the level of execution is minimized, while mutual “cross-talk” in relation to movement planning is promoted (Liu et al., 2002). The endeavor of extending this approach to humans has been facilitated in recent years through new technologies that complement and extend anatomical studies undertaken using classical post-mortem techniques and animal models. A key advantage of these new approaches is the facility to obtain measurements of structural connectivity *in-vivo*, and relate these both to indices of brain activity and to behavior.

Positioned directly below the gray matter cortex, cerebral white matter forms a dense network of communication cables that connect distant brain regions, and composes half of the human brain, a percentage much greater than in any other animal (Fields, 2008). The integrity, density and structural connectivity of the white matter pathways can be measured and imaged using diffuse tensor imaging (DTI), which allows the tracking of water diffusion in tissues in the brain, using output measures such as fractional anisotropy (FA). This quantifies the diffusion of water molecules, the movement of which are constrained by cellular structures such as the walls of the axons. Molecular motion is limited further by layers of lipidic cover that constitutes the myelin sheath. The measure thus derived is largest in regions that are assumed to be heavily myelinated or that have densely packed axons, although the precise nature of the link between FA (i.e., derived *in vivo*) and human histology (i.e., myelination) remains elusive (see Alba-Ferrara and de Erausquin, 2013, for a review). Whether the measure expresses constrained diffusion caused primarily by the structure of the axon itself, or the ultrastructure of myelin sheaths, the assumption nonetheless is that higher FA reflects greater structural connectivity.

**Figure 4:**
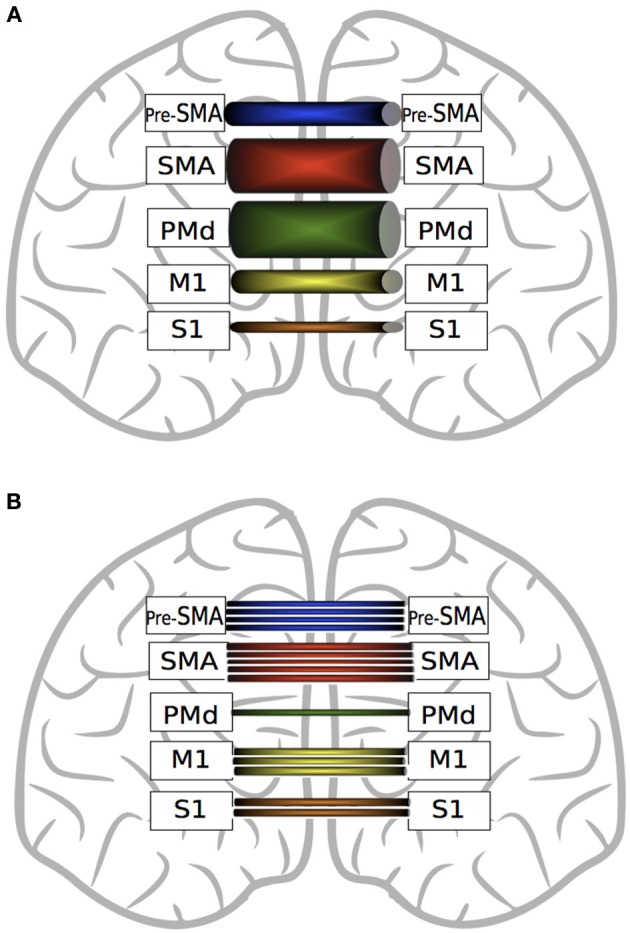
**A graphic depiction of the findings of Fling et al. (2011)**. Panel **(A)** represents fractional anisotropy (FA) of transcallosal fibers connecting homologous motor regions. Thicker tubes indicate higher FA values. SMA fibers exhibit greater FA values than pre-SMA, M1 and S1. Transcallosal PMd fiber FA values are greater than those connecting pre-SMA, M1 and S1. Fibers connecting homologous M1 and homologous pre-SMA exhibit higher FA values than S1 fibers. Panel **(B)** represents the quantity of interhemispheric fiber tracts connecting homologous motor regions. There are more fiber tracts connecting homologous SMA regions than M1, PMd and pre-SMA. There are more fibers connecting pre-SMA than M1, S1 or PMd. Transcallosal M1 fibers are more numerous than those connecting S1 or PMd. (See text for details).

While recent DTI derived evidence suggests that interhemispheric callosal projections are largely homotopic (Fling et al., 2011), within the corpus callosum there are marked differences in the quantity and strength of fibers projecting from the different components of the motor network that are involved in voluntary movement (Figure 4). In terms of quantity, there are significantly more fibers connecting homologous SMA regions than connecting M1, primary sensory cortices (S1), pre-SMA or dorsal premotor cortices (PMd) (Fling et al., 2011). In contrast to the conclusions drawn on the basis of retrograde tracing in primates, it appears that in human there are fewer interhemispheric fibers from Pre-SMA than from SMA, but more than for M1, S1 or PMd. In relation to number of fibers, there are more homotopic projections for M1 than for S1 or PMd.

It is important to note, however, that this metric is not necessarily paramount in relation to the functional implications of white matter connectivity. PMd-PMd interhemispheric connections, along with SMA-SMA fiber tracts, display the highest microstructural integrity (FA) values (Figure 3). Lower FA values are obtained for homotopic projections from S1, M1 and pre-SMA, whereas in relation to PMv, direct interhemispheric connections have not been identified using DTI (Fling et al., 2011).

Two critical considerations are thereby highlighted. In the first instance, summary measures of connectivity calculated for the entire bundle of fibers passing across the corpus callosum are unlikely to be revealing with respect to the mediation of cross education, when these are considered in relation to variations in behavioral outcomes exhibited within or between groups of individuals. Furthermore, specific metrics of fiber orientation and strength—such as FA values (derived for projections between clearly delineated nodes in the motor network), will probably bear a more direct relation to functional interactions between the limbs than global or local estimates of fiber number.

As case in point, Bonzano et al. (2011a) reported that in a group of patients with corpus callosum pathology due to multiple sclerosis (MS), levels of interlimb transfer in a reaction time task were not associated with FA values derived for the entire callosal body. A positive relationship was, however, obtained when FA values were calculated for a subregion (CC3), following de Lacoste et al. (1985), presumed to encompass fibers projecting to primary motor and sensory areas (but not for a subregion (CC2) deemed to contain fibres projecting to premotor and supplementary motor areas).

#### Structural connectivity between the primary motor areas

It has been demonstrated that the microstructural integrity of the white matter in transcallosal pathways projecting into the M1 hand area correlates positively with levels of interhemispheric inhibition, measured both using paired pulse techniques (Wahl et al., 2007), and the ipsilateral silent period (Koerte et al., 2009; Fling et al., 2011). Given that greater microstructural integrity—as indexed by FA, may be reflective of either the dense packing of many fibers, or their myelination quality (expressed as lower signal degradation), the finding of elevated interhemispheric inhibition between motor cortices in individuals with higher FA values may be indicative of an excitatory signal transmitted via the corpus callosum that results in proportionately greater activation of the inhibitory interneuron network in the target hemisphere. As emphasized previously, however, the net balance between inhibition and facilitation that results from transcallosal input (i.e., from the opposite M1) is also subject to task-dependent modulation by areas upstream of M1, such as premotor cortex, which assume a focusing role by regulating the activity of interneurons in primary motor cortex (Münchau et al., 2002).

The relevance of this general point in relation to the interpretations that might be drawn concerning the structural pathways that mediate interhemispheric inhibition on the one hand, and cross education of motor function on the other, cannot be overstated. While performing index finger to thumb opposition movements, individuals with MS exhibit higher levels of BOLD response in ipsilateral M1, and decreased levels of interhemispheric inhibition (registered using iSP). These variables correlated (negatively and positively, respectively) with FA values calculated for the body of the corpus callosum (Lenzi et al., 2007). Nonetheless, the capacity for intermanual transfer appears largely unaffected in this population (Bonzano et al., 2011a).

In a related vein, there is an age-related dissociation in the relationship between IHI and M1 callosal tract microstructural integrity. Young adults with relatively larger FA values also exhibit greater (iSP derived) interhemispheric inhibition, whereas for older adults the opposite relationship is obtained (Fling and Seidler, 2012). There is mounting evidence to suggest that both the quantity and quality of cerebral white matter diminishes with age (e.g., Seidler et al., 2010; Sullivan et al., 2010). It is also well-established that experimentally derived measures of interhemispheric inhibition diminish overall with advancing age, and that these changes are related to the level of ipsilateral activity that is present during the performance of single limb tasks (Talelli et al., 2008a,b). Furthermore, commensurate elevations of crossed facilitation (e.g., Fling and Seidler, 2012) and contralateral irradiation of motor output throughout the lifespan have been thoroughly documented (see Addamo et al., 2007 for a review). At first glance it might therefore appear paradoxical that levels of cross education are diminished in older adults in comparison to younger counterparts (Bemben and Murphy, 2001) in some cases markedly so (Hinder et al., 2011). Taken as a whole however, these lines of evidence serve to emphasize that the structural factors that directly influence levels of interhemispheric inhibition between the primary motor cortices may not to be those that assume a principal role in mediating the crossed transfer of functional capacity.

## Bilateral access approach

### Theoretical context and scope of the present analysis

**Figure 5:**
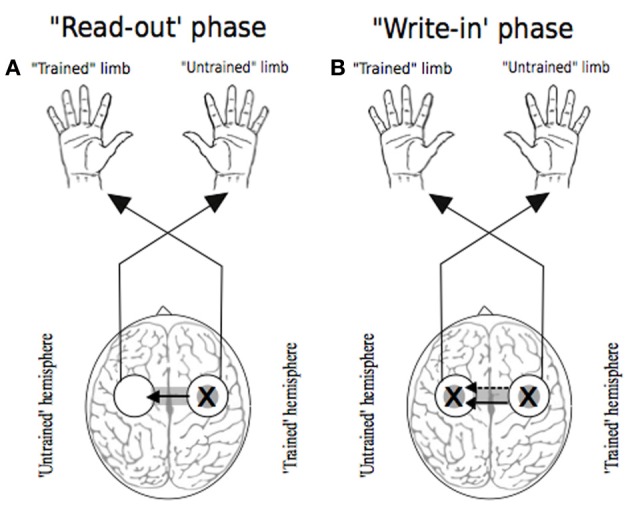
**Two phases of transfer**. In each instance the “X” represents the putative locus of training related adaptations. White circles indicate lateralized motor networks in their entirety. Following Nadel and Buresova (1968), Panel **(A)** represents the 'read-out' phase (solid arrow) whereby training related adaptations in motor networks projecting to the training limb are accessible during movements made subsequently by the untrained limb. Panel **(B)** depicts the possibility that, during the course of such access (i.e., “read out”), a “write-in” phase (dashed arrow) occurs, whereby a parallel duplicate motor engram is formed.

In seeking to account for the mechanisms that give rise to cross education of motor function, there have been numerous advocates of the view that neuroplastic changes occurring in conjunction with unilateral training are amenable to utilization (subsequently) when the untrained limb is engaged. A point of contrast with cross activation models is that task and effector specific changes in the state of neural circuits projecting to the muscles of the quiescent limb are not necessarily anticipated for the period of training. The integrity of any such distinction necessarily depends on the facility to demarcate brain regions that assume a *functional* role in relation to movements performed on one side of the body, but not on the other. As highlighted previously, it is not even clear that primary motor cortex can be categorized in this manner (Bianki and Shrammapril, 1985). Although some proponents of the bilateral access approach have emphasized the role of the corpus callosum as a means for information transfer from a single hemisphere in which the “motor engram” has been elaborated (e.g., Taylor and Heilman, 1980), it is not necessarily apparent that such lateralization is a logical necessity. On a priori grounds alone, bilateral representation (e.g., Parlow and Kinsbourne, 1989) of a capability acquired unilaterally cannot be excluded. The possibility has also been highlighted (Nadel and Buresova, 1968) that transcallosal “read-out” of a lateralized memory trace may initiate an active process in the “trained” hemisphere which precipitates transcallosal information flow in the opposite direction that is to say—from the trained to the untrained hemisphere (Figure 5). Through active “write in,” which may occur over the course of just a few trials or on even a single trial (Fenton and Bures, 1994), a duplicate “motor engram” is formed in the untrained hemisphere—a mode of transfer that has been termed *imperative*. Direct “read-out” of a lateralized engram that does not require an equivalent active process has been designated *facultative* transfer (Bureš et al., 1988).

Necessarily therefore, the patterns of neural activity that are instrumental in enhancing execution during training, and their relationship to those present during the ensuing performance of the opposite untrained limb, are an empirical matter. It is our argument that when considered on this basis, there emerge few grounds for distinction between the cross activation and bilateral access models. Rather, we contend that the degree to which there is bilateral engagement of various elements of the motor network, and the extent of cross education that accrues from unilateral training, is contingent upon specific task parameters. In seeking to illustrate this point in the sections that follow, we restrict our attention to a relatively small subset of exemplars. Consideration is not, for example, extended to interlimb transfer in the context of prism (e.g., Martin et al., 1996), visuomotor (e.g., Sainburg and Wang, 2002) or force-field adaptation (Criscimagna-Hemminger et al., 2003). In addition, factors that might influence asymmetries of transfer between the dominant and non-dominant limb are not considered at length.

Rather, we focus our attention upon variants of sequential motor tasks. Typically these require that buttons or keys be pressed by the fingers of one hand—often by means of isometric contractions, in accordance with a memorized or perceptually cued sequence. In some variants (e.g., Hicks et al., 1982), the participant is instructed to repeat the sequence as many times as possible within a fixed interval. In others, such as the serial reaction time task (SRTT), (Nissen and Bullemer, 1987), participants respond repeatedly to a fixed sequence of stimuli, which is not typically made explicit. Learning is inferred on the basis of decreases in reaction time that accord with the probabilities governing transitions between successive stimuli in the sequence. The demands associated with actuation nominally remain fixed. That is, there is no overt stipulation for the keys or buttons to be depressed with increasing force or rapidity. Thus, since it is generally assumed that such tasks place *minimal* requirements on motor execution, progressive changes in their performance are typically interpreted as evidence of motor sequence learning (Hardwick et al., 2013).

### Task parameters: a case in point

In a study that engaged a large cohort of young adults in a five-key sequential tapping task performed with either the left or the right hand, Parlow and Dewey (1991) required that a subset of participants undertake the training phase (ten 15 s trials) while simultaneously engaging the opposite (“untrained”) limb in the production of sustained (i.e., 15 s) sub-maximal (Experiment 1) or maximal (Experiment 2) isometric grip force. It was noted that these groups exhibited positive transfer of performance from the trained to the untrained limb that was comparable to that obtained for (control) groups that did not engage in the secondary task. On the basis of the assumption that the generation of maximum grip force by the “untrained” limb during practice of the sequential tapping task (i.e., by the opposite limb) prevented training task-specific “motor overflow” from engaging brain circuits that might in principle become adapted, these findings are commonly considered support for bilateral access models of cross education. Rather than it being the case that homologous circuits were engaged by this manipulation, the markedly different demands imposed by the sequence generation and isometric grip force tasks, coupled with the observation that dual task deficits were not necessarily present during acquisition, suggest an alternative interpretation—that the engagement of somewhat distinct brain circuits was required in each instance. In this light, the fact that the (secondary) task did not impede the transfer of acquired competence on all variants of the primary task reflects a *lack* of interference between the patterns of motor network activity associated with each form of action. The more general point is thereby illustrated. The specific neural pathways that mediate cross education of motor function are likely to be strongly contingent upon the dimensions of the task. These dimensions might include, for example, the degree to which maximal motor output is demanded, the extent to which the action goals can be represented in an internal or external coordinate scheme (e.g., Hikosaka et al., 1999), or the relevance of procedural knowledge (Obayashi, 2004).

### Neural correlates of sequence learning

Empirical evidence derived from functional neuroimaging has reinforced the appreciation that distributed brain networks necessarily function in concert during the learning of motor sequences. Nonetheless, it does not inevitably follow that all of the constituent regions within these networks assume an equivalent role in the mediation of cross education. In the following sub sections, we adopt a pragmatic approach, whereby the individual brain regions that may be implicated are discussed individually. This should not be taken to imply that they function in an isolated fashion. Rather, there is unquestionably an integrated dynamic interplay between these regions, with their relative contributions to cross education having more or less emphasis depending upon factors such as task type, stage of learning, and task complexity.

#### Dorsal premotor cortex

In primates, stimulation of both dorsal and ventral premotor cortices results in observable twitch responses, suggesting that they may play an instrumental role in the generation and control of movement (Dum and Strick, 2005). Furthermore, in addition to direct descending spinal projections, PMd has reciprocal connections with ipsilateral M1 (Dum and Strick, 1991, 2002; He et al., 1993, 1995), rendering it well-placed to provide neuromodulatory control of M1 output. Within PMd, there appears to be a rostrocaudal continuum of activity, with rostral (anterior) locations implicated in sensory and working memory style tasks, and caudal (posterior) aspects of PMd more prominently engaged in motor learning. More generally, given the direct projections that exist between rostral PM and prefrontal areas, and between caudal PM and M1 (and spinal cord) respectively, it has been proposed that rostral PM fields may represent a functional node of a prefrontal network, whereas caudal PM may be regarded as a true motor area engaged primarily during movement execution (Schubotz and von Cramon, 2003).

In a recent meta-analysis, Hardwick et al. (2013) reported that the degree to which dorsal premotor cortex (PMd) activity was lateralized depends critically upon the characteristics of the task. It was noted that variants of the SRTT elicited bilateral PMd activity, whereas tasks that required the learning of novel movement kinematics and dynamics gave rise primarily to left PMd activity (i.e., independently of the side of execution). This finding may be interpreted in light of the conjecture that the left PM is engaged during the acquisition of new motor patterns –whether performed with the dominant or non-dominant hand, whereas the right PM is involved to a greater degree in the storage of sequences (e.g., Schubotz and von Cramon, 2003). The latter supposition is supported by the observation that levels of right PM activation co-vary with length of motor sequence (Sadato et al., 1996). In a perceptual counterpart of the SRTT, the requirement for serial prediction *per se* was associated with elevated activity in right PM. Increases in the number of elements in the sequence resulted in prominent increases in the levels of activation registered in PMd within both hemispheres (Schubotz and von Cramon, 2002). Thus, while the task-specific factors that determine the lateralization of PMd activity remain to be resolved, there is consensus that this brain region is a pivotal structure for motor learning in general, and for motor sequence learning in particular. Additionally, given that interhemispheric PMd-PMd connections are among the strongest of all motor regions (Fling et al., 2011), it may also be implicated in the cross education of performance in this context.

#### Supplementary motor area

The finding of activity in the supplementary motor area (SMA) is common to all neuroimaging studies that have investigated SRTT learning (Hardwick et al., 2013). This is unsurprising given that which is known about the role of SMA and its interactions with adjacent cingulate motor cortical regions, both of which contribute to the initiation of voluntary movement (Deecke and Kornhuber, 1978; Hoffstaedter et al., 2012). There is also evidence that the SMA plays distinct functional roles at different times during the performance and acquisition of a new movement sequence. In the gaps between the generation of individual elements, SMA serves the function of encoding and planning the next movement in the sequence (Tanji and Shima, 1994; Gerloff et al., 1997; Shima and Tanji, 1998), whereas during the execution of these elements, SMA assumes an additional role in relation to online monitoring and control (Seitz and Roland, 1992; Tanji and Shima, 1994; Shima and Tanji, 2000; Padoa-Schioppa et al., 2002; Lee and Quessy, 2003). A task specific distinction may, however, be drawn between the two composite regions of SMA: pre-SMA and SMA proper. It has been revealed that only SMA proper is activated during tasks requiring novel movement kinematics or dynamics, whereas during variants of the SRTT, both SMA proper and pre-SMA are involved (Hardwick et al., 2013). Human neuroimaging data indicating a specific functional role for pre-SMA during variants of the SRTT are also consistent with evidence derived from single cell recordings in non-human primates (Tanji and Shima, 1994; Clower and Alexander, 1998; Shima and Tanji, 2000). Aside from sequence learning, the pre-SMA appears to have functions that are predominantly cognitive in nature, serving a minimal role in other forms of motor learning. Stimulation of SMA proper appears to enhance motor learning in a task with a sequential learning component, whereas pre-SMA stimulation has no such effect (Vollmann et al., 2012). Analogous to the rostrocaudal continuum of cognitive-motor function within the PMd, the SMA, which shares with PMd a cytoarchitecturally defined location on Broadman's area 6, is similarly subdivided, with the more rostral region (pre-SMA) assuming a role in cognitive functions, and the caudal SMA proper having undisputed motor properties (Hardwick et al., 2013).

#### Primary motor cortex

While there is widespread evidence that M1 is integral to a network of brain regions involved in the learning and retention of motor skills, the extent of its contribution varies in a task and time-dependent fashion. In the initial stages of acquiring skills for which a significant degree of cognitive involvement is required, there are relatively high levels of activity in prefrontal, bilateral sensorimotor, and parietal cortices. It has been proposed that for tasks of this nature, the initial phases of skill acquisition are mediated via regions of a cortical network specialized for executive function, motor planning/execution and the processing of somatosensory feedback, and that sub-cortical circuits in the cerebellum and basal ganglia assume a commensurately greater role as automaticity of performance is achieved (Floyer-Lea and Matthews, 2004). It is also the case that during early learning of a “fast-as-possible” ballistic motor task—for which few cognitive demands might be assumed (Rosenkranz et al., 2007; Carroll et al., 2008; Hinder et al., 2011), and in visuomotor adaptation tasks—following a perceptible state transition (Riek et al., 2012), there are increases in the excitability of corticospinal projections from M1.

The relative contribution of M1 to the most rapid phase of performance adaptation, as opposed a slow repetition-dependent component, in tasks requiring modified movement kinematics or dynamics, and the significance of this demarcation with respect to acquisition and retention, remains a matter of considerable debate (e.g., Richardson et al., 2006; Galea et al., 2011; Orban de Xivry et al., 2011; Riek et al., 2012). During unilateral motor sequence learning, elevated activity is registered in M1 ipsilateral to the training limb (Daselaar et al., 2003; Bischoff-Grethe et al., 2004; Verstynen et al., 2005). This is thought more likely to be reflective of excitatory rather than inhibitory neural activity (Waldvogel et al., 2000). There is also some evidence to suggest that left M1 is activated regardless of the limb that is the focus of training. In contrast, right M1 is not engaged prominently during right hand execution (Hardwick et al., 2013). While this pattern suggests that the left primary motor cortex performs a specialized function in this form of task (Jueptner et al., 1997b; Seidler et al., 2005; Bapi et al., 2006), it appears likely that the activity is more closely related to effector aspects than to serial prediction *per se* (Sanes and Donoghue, 2000; Hardwick et al., 2013).

#### Superior parietal lobule

The parietal cortex has traditionally been considered as the bridge between vision and movement (Critchley, 1953; Milner and Goodale, 1993), with the superior parietal lobule (SPL) in particular assuming a significant role in relation to actions involving the hands (Mountcastle et al., 1975; Rizzolatti et al., 1998; Connolly et al., 2003; Glover et al., 2005; Battaglia-Mayer et al., 2007). This area is activated consistently during all motor variants of the SRTT, but not necessarily in tasks that require the acquisition of novel limb kinematics or dynamics. As most often the SRTT includes the requirement to respond to visual stimuli, and given the centrality of its relationship with PMd in visuomotor integration and control (Wise et al., 1997), it is perhaps unsurprising that the SPL is engaged during this type of motor sequence learning. In a learning task in which an auditory metronome was used to pace movements, and visual feedback was not provided, significant levels of SPL activation were not obtained (Jantzen et al., 2002). In the context of a network in which PMd represents the “hub” of sequence learning, the SPL thus appears to perform a relatively specific role in the transformation of sensory input into motor output (e.g., Hardwick et al. (2013).

#### Thalamus, striatum, and cerebellum

The role of the striatum has been emphasized as a critical component for the planning, acquisition and execution of new motor skills (Doyon et al., 2009). It receives major afferent inputs from cortical areas, from the midbrain, and from the thalamus (Delong and Wichmann, 2007). Its principal role is thought to lie in encoding motor programs, and it is activated consistently during both implicit and explicit sequence learning (see Doyon et al., 2009 for a review). Neuroimaging data suggests that there exists a dynamic functional interplay between the striatum and cerebellum while subjects are acquiring a motor skill—up to the point of asymptotic levels of performance. Once the behavior is extremely well-learned, activity in the cerebellum becomes barely detectable (Friston et al., 1992; Grafton et al., 1994; Seitz et al., 1994; Jueptner et al., 1997a; Doyon et al., 2002), whereas activation in the striatum persists (Grafton et al., 1994; Doyon et al., 1996; Jueptner et al., 1997b). This has led to the view that striatal activity is associated with the long-term retention of motor skill. Similarly the thalamus: a multi-nucleus “relay station,” receiving inputs from an array of brain sub-systems, and conducting them onwards to their appropriate destinations, also shows “sustained” activation after asymptotic levels of performance have been achieved (Duff et al., 2007). In the SRTT studies assessed by Hardwick et al. (2013) in their recent meta-analysis, the (left) thalamus was prominently engaged, an effect that was most apparent when in contrast with tasks that require the acquisition of novel dynamics or kinematics.

One of the key roles ascribed to the cerebellum in motor learning is that of “state estimation,” whereby the actual sensory consequences of actions are compared to the predicted sensory consequences. It is upon the basis of the prediction errors thus derived that improvements of performance, in relation to parameters such as speed and accuracy, are thought to develop (Manto et al., 1994; Miall et al., 2007; Tseng et al., 2007; Miall and King, 2008). As SRTT variants have actuation demands that nominally remain fixed, it is perhaps not surprising that this form of task acquisition is associated with lower levels of cerebellar activity than other forms of motor learning. With respect to regional variation, engagement of the right lateral cerebellum in the SRTT appears to be a consistent finding (Hardwick et al., 2013).

### Bilateral transfer of sequence learning—functional brain imaging

Very few empirical studies have used neuroimaging techniques to investigate bilateral transfer of sequence learning. Perez et al. (2007a) reported that following right limb SRTT training, areas of (fMRI registered) activation during left hand task execution included bilateral SMA, PMd, striatum, extrastriate visual cortex, cerebellum, thalamus, and also the right M1. It should be noted in this context that as projections to the cerebellum are double-crossed, activation registered in this region is generally associated with movements of the ipsilateral limb. Additionally, activity in some regions was correlated with behavioral measures of intermanual transfer of performance. Pre-training activity in the right ventrolateral posterior (VLp) thalamic nucleus was predictive of the amount of interlimb transfer that would be observed following training, and post-training activity in the (bilateral) ventrolateral anterior (VLa) thalamic nucleus and SMA correlated positively with the amount of interlimb transfer that had occurred. Importantly, activity in these areas was not correlated with performance changes in a control movement sequence.

The areas of activation detected in sequence learning tasks depend, at least in part, upon whether the transfer task requires that the sequence is executed in the original spatial format (i.e., defined with respect to an external coordinate scheme) or in a mirrored layout (that preserves the internal (anatomical) coordinate mapping). Instances of the latter type would require the use of the corresponding effectors on the opposite (untrained) hand, and generation for the homologous muscles of the same motor output patterns as those that required during training. In the case of handwriting, similar patterns of brain activation are noted in right-handed subjects when the right hand is writing normally, and the left hand is required to write in a mirrored format. Many additional brain regions are, however, engaged when the left hand is required to write such that the “normal,” (with respect to the right hand) spatial pattern is preserved, presumably as additional transformations are required to generate the novel muscle synergies (Halsband and Lange, 2006). In this task context at least, the paucity of additional brain activity suggests that mirrored performance by the untrained limb is subserved by the same engram that is utilized by the trained limb (Grafton et al., 2002).

### Bilateral transfer of sequence learning—electrophysiological indices

Although neuroimaging techniques are invaluable for localizing variations in cerebrovascular demand, they cannot be used easily to assess inhibitory neural processes (Waldvogel et al., 2000). We have highlighted previously that for tasks that require maximum levels of motor output, these processes may assume a functional role in relation to cross education. Is there evidence that they are implicated in bilateral transfer of sequence learning? Perez et al. (2007b) reported that following unilateral SRTT training, there was a decrease in IHI from the M1 contralateral to the training limb, to the M1 contralateral to the transfer limb. The extent of this decrease was correlated with the amount of non-specific performance transfer to the untrained limb. In the SRTT, this is typically expressed as decreased reaction times in all aspects of the task, including random blocks that have no sequential component (Robertson, 2007). The level of sequence-specific transfer of learning was not, however, correlated with IHI measures. This pattern of outcomes accords with that reported by Hortobagyi et al. (2011), and suggests that the non-specific transfer observed for the SRTT may be similar in nature to the cross education observed for maximal output training tasks. The results of Perez et al. (2007b) further imply that experimentally derived measures of interhemispheric inhibition between the primary motor cortices are insensitive to the neural adaptations that mediate the interlimb transfer of elements specific to sequence learning. Following SRTT training, SICI is reduced in both the trained and untrained M1, a finding that is consistent with the proposal (Bianki and Makarova, 1980) that a narrowing of excitatory focus in the primary motor cortex contralateral to the training limb emerges from reciprocal interhemispheric interactions. Furthermore, the observation that elevations in the net excitability of corticospinal projections from M1 were present only for the hemisphere contralateral to the training limb (Perez et al., 2007b), is consistent with the conclusion highlighted previously that the functional adaptations that underpin cross education are either mediated by interneuronal networks within primary motor cortex- other than those directly engaged in generating corticospinal output, or via changes in the effectiveness of synaptic transmission through projections from other areas of the motor network onto M1 targets. These two possibilities are not exclusive.

Various forms of non-invasive brain stimulation, including TMS, have been used to disrupt processing in a region of interest during classical motor learning tasks. In noting any associated behavioral effects, the usual intent is to draw causal inferences. An important caveat holds, however, when M1, or indeed any other area having descending projections to the spinal cord, is the region of interest. The motor system may accommodate this challenge by altering the activity in other brain areas involved in movement planning and execution in a manner that preserves motor output (e.g., Touge et al., 2001; Shemmell et al., 2007; Ortu et al., 2009). Thus, it may not be possible to determine whether the effect of M1 stimulation upon motor learning is attributable to an altered contribution of the target region, or due to compensatory changes occurring elsewhere. The problem is particularly acute when a limited range of measures is employed to assess the impact of the intervention. For example, in a recent investigation, Riek et al. (2012) demonstrated that following the administration of theta burst rTMS prior to initial learning in a visuomotor adaptation task, the overt characteristics of performance (as assessed by trajectory error and movement time) were maintained. There was, however, a profound impact upon the latency of response preparation—a measure not obtained typically in adaptation paradigms. There are more general implications. The brain region that is of critical functional importance in relation to the behavior under consideration may be one that receives (excitatory or inhibitory) inputs from the stimulation target. Thus, the effects of such interventions upon learning can rarely be considered profitably without additional controls, and corroborating evidence derived from other investigative techniques.

With these qualifications in mind, we turn to one of the few studies in which this general approach has been applied to investigate the contribution of a specific region—in this case SMA, to intermanual transfer. As aforementioned, (see section Supplementary Motor Area), the contribution of SMA to sequence learning is thought to be phase dependent. It is engaged in encoding and planning the next movement in a sequence, and in controlling and monitoring movements once they are initiated. Perez et al. (2008) reported that in a SRTT variant, applying 1 Hz rTMS to SMA along the sagittal midline in the intervals between successive movements reduced levels of intermanual transfer. Conversely, applying rTMS to SMA during movement execution had no such effect. On this basis, the authors concluded that the contribution of SMA to the interlimb transfer of sequence learning occurs primarily in the intervals between movements (Perez et al., 2008). Given the poor temporal resolution of fMRI and PET, which hampers the use of corresponding experimental (i.e., imaging) designs, it is difficult to determine the degree to which a mediating role of SMA, as opposed to pre-SMA is implied by these data. In light of the considerations noted above, it may be noted that the midline SMA stimulation had no impact upon the rate at which performance improved for the training limb. It has been suggested that this may indicate that distinct mechanisms mediate the increases in performance manifested by the training limb, and the concurrent increase in capability exhibited by the opposite limb (Perez et al., 2008).

### Bilateral transfer of sequence learning—structural correlates

White matter structural integrity is thought to impinge directly upon motor performance, as the quality of myelin and axon diameter impact upon the propagation speed of neural impulses (Fields, 2011). These in turn contribute to the larger scale synchronization of signals across distributed components of the functional networks that are required for skilled task execution and learning (Fields, 2008). In the present context, interhemispheric callosal pathways and intrahemispheric association fibers are of particular interest.

Neuroimaging studies have demonstrated that a distributed network of frontal, parietal and motor regions, are activated intrahemispherically during explicit (visuomotor) sequence learning (Jenkins et al., 1994; Schlaug et al., 1994; Honda et al., 1998; Sakai et al., 1998). The superior longitudinal fasciculous (SLF) is a pair of fiber bundles that connects these regions *intra*hemispherically (Makris et al., 2005; Koch et al., 2010), and provides the structural basis for their interaction. Even when training is unilateral, there appears to be bilateral engagement of this network (Honda et al., 1998; Müller et al., 2002), thus suggesting that the fibers of the corpus callosum are also essential for this type of motor learning to proceed. In accordance with this view, Bonzano et al. (2011b) reported that the integrity of transcallosal fibers had a much greater bearing on an individual's capacity for unilateral (explicit) sequence specific learning, than similar indices derived for the fibers of the SLF. No such association was found for non-specific sequence learning (i.e., a decrease in reaction time obtained when stimuli are presented randomly). These outcomes imply that the involvement of transcallosal pathways is crucial, at least for this form of sequence learning.

**Figure 6:**
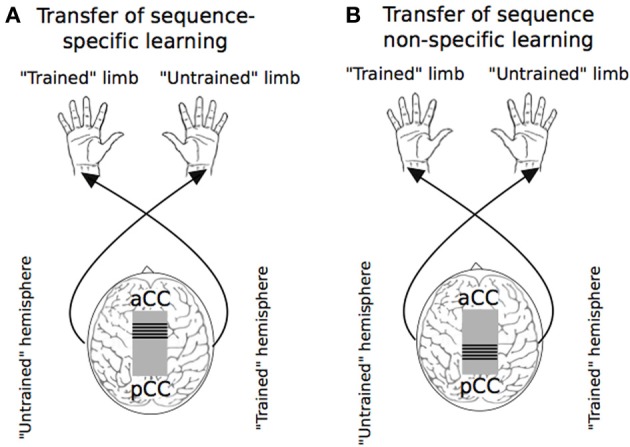
**Callosal pathways mediating the transfer of sequence specific and sequence non-specific learning in the Serial Reaction Time Task**. “aCC” and “pCC” define the anterior-posterior axis of the corpus callosum. Panel **(A)** illustrates that the transfer of sequence-specific aspects of the SRTT occurs primarily via interhemispheric fibers in the anterior midbody of the corpus callosum. Panel **(B)** indicates that the transfer of non sequence-specific learning is predominantly via fibers in the posterior midbody of the corpus callosum.

It is almost certainly the case that distinct sub-portions of the corpus callosum subserve different functions. The degree to which there is interlimb transfer of non-specific learning in a SRTT context, correlates positively with fractional anisotropy (FA) values for the posterior midbody of the corpus callosum (Bonzano et al., 2011a). This may accord with the finding of Perez et al. (2007b) that variations in IHI are related specifically to disparities in the transfer of non-specific motor sequence learning. The differentiated roles of the corpus callosum in relation to cross education in this class of tasks is further emphasized by findings that anterior callosotomy produces deficits in intermanual transfer in circumstances in which sequence-specific learning is exhibited by the training limb (de Guise et al., 1999; Peltier et al., 2012). In suggesting that the anterior body of the corpus callosum is essential for the effective transfer of sequence specific motor learning, the outcomes are complementary to those showing that microstructural characteristics of the posterior midbody of the corpus callosum determine levels of transfer of non sequence-specific learning (Bonzano et al., 2011a) (Figure 6). They are also consistent with the more general assumption that these facets of SRTT learning are processed by different brain networks (Hikosaka et al., 1999; Bischoff-Grethe et al., 2004). Fibers passing through the posterior midbody of the corpus callosum may mediate transfer of non sequence-specific learning, whereas, interhemispheric projections between homologous regions of SMA appear a more likely waypoint for transfer of sequence specific learning. Transcallosal SMA-SMA connections are more plentiful and have greater structural integrity than those connecting any other motor region (Fling et al., 2011), however, we are not aware of any direct investigation of the relationship between the structural characteristics of transcallosal SMA projections and expressions of cross education.

## Conclusions

The credo motivating the present review is that the transfer of strength or vigor accruing from a bout of unilateral resistance or ballistic training, and the transfer of skill following a period of unilateral skill training are mediated by common mechanisms. In seeking to illustrate the origins of this conviction, we elected to present empirical findings principally in the context of the experimental paradigms in which they were derived. This approach was driven by a number of key considerations. The relevant research literature is fragmented. There are remarkably few instances in which the engagement of specific neural pathways has been studied by applying the same analytic approach to multiple paradigms that bring forth the expression of cross education. Similarly, it has seldom been the case that the dimensions of single tasks have been manipulated systematically with a view to altering the level of cross education. By and large therefore, the necessary inferences cannot be drawn directly. The intercession of common mechanisms can, however, be deduced through synthesis and assimilation. In the preceding sections we have sought to highlight the findings that are critical in relation to this integration. In these closing sections, we provide an explicit summary of that which can reasonably be concluded as a consequence, and present a number of conjectures—for which resolution may await experimental designs beyond those that have thus far been customary in the study of cross education.

### Summary

During the course of unilateral training, both in tasks that demand maximal levels of motor output, and in those that require the learning of action sequences, there is augmentation of activity (registered by neuroimaging) in the primary motor cortex ipsilateral to the training limb (e.g., Dai et al., 2001), and an accompanying elevation in the excitability of corticospinal output projections, as revealed by increases in the amplitude of motor potentials evoked by TMS. The latter changes persist beyond the cessation of training, and extend beyond homologues of the muscles engaged in the training task (Carroll et al., 2008). With respect to both indices, the extent of the induced variation is contingent upon the level of efferent drive required to perform the training movements. This factor is also a determining influence on the level of cross education exhibited by the opposite (“untrained”) limb. Nonetheless, there is no apparent association between the excitability of corticospinal output pathways projecting to the untrained limb—when these are assessed at rest, and the level of contemporaneous (i.e., acute) or deferred (i.e., chronic) transfer of performance (e.g., Hinder et al., 2011; Hortobagyi et al., 2011). At least two possibilities are thus admitted. Cross education of motor function may be mediated by mechanisms acting via neural pathways projecting from areas upstream of the primary motor cortices. If this is the case, the elevations in the excitability of corticospinal projections observed during and immediately following training of the opposite limb, may simply reflect crossed facilitation that is not instrumentally related to transfer of performance. An alternative and not exclusive possibility is that cross education effects are mediated, at least in part, by adaptations in interneuronal networks within M1 other than those directly engaged in generating corticospinal output. In this conception, changes in the state of these interneuronal networks, which may play a role in narrowing of the excitatory focus of motor output, will be expressed in circumstances in which they receive synaptic drive, but not necessarily when the output circuits of the primary motor cortex are activated by low intensity single pulse TMS. In this regard, synaptic drive to these networks may occur not only during voluntary contractions, but also in non-physiological contexts, including paired pulse TMS paradigms, such as those employed to obtain measures of interhemispheric inhibition. It is therefore notable that decreases in IHI (“trained” to “untrained” hemisphere) are expressed acutely during the course of a single unimanual training session and chronically over multiple sessions, and that these changes can be related to the degree of cross education (Hortobagyi et al., 2011). It is likely that such decreases in IHI express alterations in the excitatory-inhibitory balance within interneuron circuits local to the hemisphere ipsilateral to the training limb, rather than adaptive changes in the characteristics of the transcallosal volley.

These considerations in relation to M1 notwithstanding, evidence derived from the functional and structural neuroimaging literature suggests that there is greater scope for inter-hemispheric interactions between other elements of the motor network during the production of unilateral movements. On an a priori basis alone, it would appear likely that both in the context of tasks that require maximal levels of motor output, and in those emphasizing the learning of action sequences, there are demands imposed upon the functional capacities of specific brain regions that will be subject to adaptive pressure during training that brings about marked improvements in performance. For example, the cingulate motor area (CMA), which is strategic entry point for limbic influence on the voluntary motor system, is closely associated with the amount of effort demanded by a motor task (Winterer et al., 2002), and exhibits activity that is modulated when the ipsilateral hand is engaged (Kermadi et al., 2000). Involvement of the SMA would be anticipated in tasks that impose requirements for movement planning, both in relation to the totality of an action sequence, and with respect to the individual elements of that sequence. Similarly, the dorsal premotor cortex (PMd) is a pivotal structure for motor learning in general, and for motor sequence learning in particular. The relative contributions of these regions will vary not only in accordance with specific task parameters, but also over time as the adaptations that form the basis of cross education are initiated and consolidated. In so much as activity in the striatum (e.g., Doyon et al., 1996) and thalamus (e.g., Duff et al., 2007) exhibit sustained activation after asymptotic levels of performance have been achieved, it is probable that these regions are associated with the long-term persistence of the transfer effects.

### Conjectures

Following Bianki (e.g., Bianki and Makarova, 1980; Bianki and Shrammapril, 1985), we propose that reciprocal interactions between the primary motor cortices are an obligatory facet of unilateral training, and that these serve to narrow the excitatory focus of cortical output to the principal muscles engaged in the task via modifications of surround inhibition. In addition to being specific to homologues of the muscles engaged in training, the concurrent and contingent adaptations induced ipsilateral to the training limb are functional rather than incidental. While the surround inhibition is instantiated in circuits local to M1, its modulation during training is mediated by inputs from other nodes of the motor network. Synaptic drive directed subsequently to these adapted circuits results in patterns of efference characterized by greater specificity in recruitment of the focal muscles engaged in a task, and in disengagement of muscles with actions that might otherwise interfere with the desired movement action. To the extent that the remodeling of motor output resembles that which is exhibited by the trained limb, cross education will be demonstrated. Necessarily the relative contribution of this mechanism to the behavioral effects will be greatest in those tasks for which enhancements in performance outcomes do not require the composition of novel synergies or the execution of novel action sequences.

Similarly, the comparative contributions of inter-hemispheric interactions between other elements of the motor network to the interlimb transfer of performance are task dependent. In circumstances in which increased effort or volition results in superior execution of the training movements, bilateral adaptations in neural circuits receiving projections from the cingulate motor areas, including targets within M1, are to be anticipated. Variations with respect to other (orthogonal) task dimensions, such as the requirement that new activation profiles be generated in refashioning muscle synergies, or that a fixed sequence of actions be reproduced, will lead to consequential changes in the state of projections from disparate regions of the network. Common to all such task-contingent variations is their consolidation over the course of extended training in thalamic and striatal relays, thereby providing the substrate for retention of cross education.

### Conflict of interest statement

The authors declare that the research was conducted in the absence of any commercial or financial relationships that could be construed as a potential conflict of interest.

## References

[B1a] Addamo P. K. Farrow M. Hoy K. E. Bradshaw J. L. Georgiou-Karistianis N. (2007). The effects of age and attention on motor overflow production-A review. Brain Res. Rev. 54, 189–204 10.1016/j.brainresrev.2007.01.004 17300842

[B1] Alba-Ferrara L. M. de Erausquin G. A. (2013). What does anisotropy measure? Insights from increased and decreased anisotropy in selective fiber tracts in schizophrenia. Front. Integr. Neurosci. 7:9 10.3389/fnint.2013.00009 23483798 PMC3593197

[B2] Anguera J. A. Russell C. A. Noll D. C. Seidler R. D. (2007). Neural correlates associated with intermanual transfer of sensorimotor adaptation. Brain Res. 1185, 136–151 10.1016/j.brainres.2007.09.088 17996854

[B3] Arányi Z. Rösler K. M. (2002). Effort-induced mirror movements. Exp. Brain Res. 145, 76–82 10.1007/s00221-002-1101-1 12070747

[B4] Archontides C. Fazey J. A. (1993). Inter-limb interactions and constraints in the expression of maximum force: a review, some implications and suggested underlying mechanisms. J. Sports Sci. 11, 145–158 10.1080/02640419308729978 8497017

[B5] Asanuma H. Okuda O. (1962). Effects of transcallosal volleys on pyramidal tract cell activity of cat. J. Neurophysiol. 25, 198–208 13862744 10.1152/jn.1962.25.2.198

[B6] Avanzino L. Teo J. T. H. Rothwell J. C. (2007). Intracortical circuits modulate transcallosal inhibition in humans. J. Physiol. 583, 99–114 10.1113/jphysiol.2007.134510 17556392 PMC2277247

[B7] Bapi R. S. Miyapuram K. P. Graydon F. X. Doya K. (2006). fMRI investigation of cortical and subcortical networks in the learning of abstract and effector-specific representations of motor sequences. Neuroimage 32, 714–727 10.1016/j.neuroimage.2006.04.205 16798015

[B8] Battaglia-Mayer A. Mascaro M. Caminiti R. (2007). Temporal evolution and strength of neural activity in parietal cortex during eye and hand movements. Cereb. Cortex 17, 1350–1363 10.1093/cercor/bhl046 16920885

[B9] Baumer T. (2006). Magnetic stimulation of human premotor or motor cortex produces interhemispheric facilitation through distinct pathways. J. Physiol. 572, 857–868 16497712 10.1113/jphysiol.2006.104901 PMC1780006

[B10] Beck S. Hallett M. (2011). Surround inhibition in the motor system. Exp. Brain Res. 210, 165–172 10.1007/s00221-011-2610-6 21424259 PMC4158822

[B11] Bemben M. G. Murphy R. E. (2001). Age related neural adaptation following short term resistance training in women. J. Sports Med. Phys. Fitness 41, 291–299 11533557

[B12] Berlucchi G. Boller F. Grafman J. (1990). Commisurotomy studies in animals, in Handbook of Neuropsychology, Vol. 4, eds Boller F. Grafman J. (Amsterdam: Elsevier), 9–47

[B13] Bianki V. L. (1981). Interaction between transcallosal and thalamocortical excitation. Neurosci. Behav. Physiol. 11, 328–335 10.1007/BF01184194 6283417

[B14] Bianki V. L. Makarova I. A. (1980). Transcallosal modulation of a focus of maximal activity in the motor cortex. Neurosci. Behav. Physiol. 10, 76–82 10.1007/BF01182240 7383329

[B15] Bianki V. L. Shrammapril V. A. (1985). New evidence on the callosal system. Int. J. Neurosci. 25, 175–193 10.3109/00207458508985370 3980151

[B16] Bischoff-Grethe A. Goedert K. M. Willingham D. T. Grafton S. T. (2004). Neural substrates of response-based sequence learning using fMRI. J. Cogn. Neurosci. 16, 127–138 10.1162/089892904322755610 15006042

[B17] Bologna M. Caronni A. Berardelli A. Rothwell J. C. (2012). Practice-related reduction of electromyographic mirroring activity depends on basal levels of interhemispheric inhibition. Eur. J. Neurosci. 36, 3749–3757 10.1111/ejn.12009 23033874

[B18] Bonzano L. Tacchino A. Roccatagliata L. Mancardi G. L. Abbruzzese G. Bove M. (2011a). Structural integrity of callosal midbody influences intermanual transfer in a motor reaction-time task. Hum. Brain Mapp. 32, 218–228 10.1002/hbm.21011 20336657 PMC6870465

[B19] Bonzano L. Tacchino A. Roccatagliata L. Sormani M. P. Mancardi G. L. Bove M. (2011b). Impairment in explicit visuomotor sequence learning is related to loss of microstructural integrity of the corpus callosum in multiple sclerosis patients with minimal disability. Neuroimage 57, 495–501 10.1016/j.neuroimage.2011.04.037 21549844

[B20] Bryan W. L. (1892). On the development of voluntary motor ability. Am. J. Psychol. 5, 125–204 10.2307/1410865

[B21] Bureš J. Burešová O. Křivánek J. (1988). Brain and Behavior: Paradigms for Research in Neural Mechanisms. Chichester: John Wiley and Sons

[B22] Bütefisch C. M. Boroojerdi B. Chen R. Battaglia F. Hallett M. (2005). Task-dependent intracortical inhibition is impaired in focal hand dystonia. Mov. Disord. 20, 545–551 10.1002/mds.20367 15641012 PMC1457024

[B23] Camus M. Ragert P. Vandermeeren Y. Cohen L. G. (2009). Mechanisms controlling motor output to a transfer hand after learning a sequential pinch force skill with the opposite hand. Clin. Neurophysiol. 120, 1859–1865 10.1016/j.clinph.2009.08.013 19766535 PMC2767461

[B24] Carroll T. J. Barton J. Hsu M. Lee M. (2009). The effect of strength training on the force of twitches evoked by corticospinal stimulation in humans. Acta Physiol. (Oxf.) 197, 161–173 10.1111/j.1748-1716.2009.01992.x 19392872

[B25] Carroll T. J. Lee M. Hsu M. Sayde J. (2008). Unilateral practice of a ballistic movement causes bilateral increases in performance and corticospinal excitability. J. Appl. Physiol. 104, 1656–1664 10.1152/japplphysiol.01351.2007 18403447

[B26] Carroll T. J. Riek S. Carson R. G. (2002). The sites of neural adaptation induced by resistance training in humans. J. Physiol. 544, 641–652 10.1113/jphysiol.2002.024463 12381833 PMC2290590

[B27a] Carson R. G. (2005). Neural pathways mediating bilateral interactions between the upper limbs. Brain Res. Brain Res. Rev. 49, 641–662 10.1016/j.brainresrev.2005.03.005 15904971

[B28] Carson R. G. (2006). Changes in muscle coordination with training. J. Appl. Physiol. 101, 1506–1513 10.1152/japplphysiol.00544.2006 16888047

[B29] Carson R. G. Kennedy N. C. Linden M. A. Britton L. (2008). Muscle-specific variations in use-dependent crossed-facilitation of corticospinal pathways mediated by transcranial direct current (DC) stimulation. Neurosci. Lett. 441, 153–157 10.1016/j.neulet.2008.06.041 18582535

[B30] Carson R. G. Riek S. Bawa P. (1999). Electromyographic activity, H-reflex modulation and corticospinal input to forearm motoneurones during active and passive rhythmic movements. Hum. Mov. Sci. 18, 307–343 10.1016/S0167-9457(99)00013-5

[B27] Carson R. G. Riek S. Mackey D. C. Meichenbaum D. P. Willms K. Forner M. (2004). Excitability changes in human forearm corticospinal projections and spinal reflex pathways during rhythmic voluntary movement of the opposite limb. J. Physiol. 560, 929–940 10.1113/jphysiol.2004.069088 15331684 PMC1665277

[B31] Carson R. G. Ruddy K. L. (2012). Vision modulates corticospinal suppression in a functionally specific manner during movement of the opposite limb. J. Neurosci. 32, 646–652 10.1523/JNEUROSCI.4435-11.2012 22238100 PMC6621083

[B32] Carson R. G. Welsh T. N. Pamblanco-Valero M.-A. (2005). Visual feedback alters the variations in corticospinal excitability that arise from rhythmic movements of the opposite limb. Exp. Brain Res. 161, 325–334 10.1007/s00221-004-2076-x 15517219

[B33] Cernacek J. (1961). Contralateral motor irradiation–cerebral dominance. Its changes in hemiparesis. Arch. Neurol. 4, 165–172 10.1001/archneur.1961.00450080047005 13691977

[B34] Chefer S. I. Talan M. I. Engel B. T. (1997). Central neural correlates of learned heart rate control during exercise: central command demystified. J. Appl. Physiol. 83, 1448–1453 9375304 10.1152/jappl.1997.83.5.1448

[B35] Chen R. Yung D. Li J. Y. (2003). Organization of ipsilateral excitatory and inhibitory pathways in the human motor cortex. J. Neurophysiol. 89, 1256–1264 10.1152/jn.00950.2002 12611955

[B36] Chiou S.-Y. Wang R.-Y. Liao K.-K. Wu Y.-T. Lu C.-F. Yang Y.-R. (2013). Co-activation of primary motor cortex ipsilateral to muscles contracting in a unilateral motor task. Clin. Neurophysiol. 124, 1353–1363 10.1016/j.clinph.2013.02.001 23478202

[B37] Cisek P. Crammond D. J. Kalaska J. F. (2003). Neural activity in primary motor and dorsal premotor cortex in reaching tasks with the contralateral versus ipsilateral arm. J. Neurophysiol. 89, 922–942 10.1152/jn.00607.2002 12574469

[B38] Clower W. T. Alexander G. E. (1998). Movement sequence-related activity reflecting numerical order of components in supplementary and presupplementary motor areas. J. Neurophysiol. 80, 1562–1566 9744961 10.1152/jn.1998.80.3.1562

[B39] Connolly J. D. Andersen R. A. Goodale M. A. (2003). FMRI evidence for a “parietal reach region,” in the human brain. Exp. Brain Res. 153, 140–145 10.1007/s00221-003-1587-1 12955383

[B40] Cramer S. C. Finklestein S. P. Schaechter J. D. Bush G. Rosen B. R. (1999). Activation of distinct motor cortex regions during ipsilateral and contralateral finger movements. J. Neurophysiol. 81, 383–387 9914297 10.1152/jn.1999.81.1.383

[B41] Craske B. Craske J. D. (1986). Oscillator mechanisms in the human motor system: investigating their properties using the aftercontraction effect. J. Mot. Behav. 18, 117–145 15136276 10.1080/00222895.1986.10735374

[B42] Criscimagna-Hemminger S. E. Donchin O. Gazzaniga M. S. Shadmehr R. (2003). Learned dynamics of reaching movements generalize from dominant to nondominant arm. J. Neurophysiol. 89, 168–17 10.1152/jn.00622.2002 12522169

[B43] Critchley M. (1953). The Parietal Lobes. Oxford, England: Williams and Wilkins

[B44] Dai T. H. Liu J. Z. Sahgal V. Brown R. W. Yue G. H. (2001). Relationship between muscle output and functional MRI-measured brain activation. Exp. Brain Res. 140, 290–300 10.1007/s002210100815 11681304

[B45] Daselaar S. M. Rombouts S. A. R. B. Veltman D. J. Raaijmakers J. G. W. Jonker C. (2003). Similar network activated by young and old adults during the acquisition of a motor sequence. Neurobiol. Aging 24, 1013–1019 10.1016/S0197-4580(03)00030-7 12928061

[B46] Daskalakis Z. J. Christensen B. K. Fitzgerald P. B. Roshan L. Chen R. (2002). The mechanisms of interhemispheric inhibition in the human motor cortex. J. Physiol. 543, 317–326 10.1113/jphysiol.2002.017673 12181302 PMC2290496

[B47] Davis W. (1898). Researches in cross-education. (E. W. Scripture, Ed.). Stud. Yale Psychol. Lab. 6, 6–50

[B48] Deecke L. Kornhuber H. H. (1978). An electrical sign of participation of the mesial “supplementary” motor cortex in human voluntary finger movement. Brain Res. 159, 473–476 10.1016/0006-8993(78)90561-9 728816

[B49] de Guise E. del Pesce M. Foschi N. Quattrini A. Papo I. Lassonde M. (1999). Callosal and cortical contribution to procedural learning. Brain 122(Pt 6), 1049–1062 10.1093/brain/122.6.1049 10356058

[B50] de Lacoste M. C. Kirkpatrick J. B. Ross E. D. (1985). Topography of the human corpus callosum. J. Neuropathol. Exp. Neurol. 44, 578–591 10.1097/00005072-198511000-00004 4056827

[B51] Delong M. R. Wichmann T. (2007). Circuits and circuit disorders of the basal ganglia. Arch. Neurol. 64, 20–24 10.1001/archneur.64.1.20 17210805

[B52] Denny-Brown D. (1967). The fundamental organization of motor behavior, in Neurophysiological Basis of Normal and Abnormal Motor Acrioities, eds. Yahr M. Purpura D. (New York, NY: Raven Press), 415–442

[B53] Dettmers C. Lemon R. N. Stephan K. M. Fink G. R. Frackowiak R. S. (1996). Cerebral activation during the exertion of sustained static force in man. Neuroreport 7, 2103–2110 10.1097/00001756-199609020-00008 8930968

[B54] Diedrichsen J. Wiestler T. Krakauer J. W. (2013). Two Distinct Ipsilateral Cortical Representations for Individuated Finger Movements. Cereb. Cortex 23, 1362–1377 10.1093/cercor/bhs120 22610393 PMC3643717

[B55] Doyon J. Bellec P. Amsel R. Penhune V. Monchi O. Carrier J. (2009). Contributions of the basal ganglia and functionally related brain structures to motor learning. Behav. Brain Res. 199, 61–75 10.1016/j.bbr.2008.11.012 19061920

[B56] Doyon J. Owen A. M. Petrides M. Sziklas V. Evans A. C. (1996). Functional anatomy of visuomotor skill learning in human subjects examined with positron emission tomography. Eur. J. Neurosci. 8, 637–648 10.1111/j.1460-9568.1996.tb01249.x 9081615

[B57] Doyon J. Song A. W. Karni A. Lalonde F. Adams M. M. Ungerleider L. G. (2002). Experience-dependent changes in cerebellar contributions to motor sequence learning. Proc. Natl. Acad. Sci. U.S.A. 99, 1017–1022 10.1073/pnas.022615199 11805340 PMC117423

[B58] Duff E. Xiong J. Wang B. Cunnington R. Fox P. Egan G. (2007). Complex spatio-temporal dynamics of fMRI BOLD: a study of motor learning. Neuroimage 34, 156–168 10.1016/j.neuroimage.2006.09.006 17081770 PMC1810348

[B59] Dum R. P. Strick P. L. (1991). The origin of corticospinal projections from the premotor areas in the frontal lobe. J. Neurosci. 11, 667–689 1705965 10.1523/JNEUROSCI.11-03-00667.1991 PMC6575356

[B60] Dum R. P. Strick P. L. (2002). Motor areas in the frontal lobe of the primate. Physiol. Behav. 77, 677–682 10.1016/S0031-9384(02)00929-0 12527018

[B61] Dum R. P. Strick P. L. (2005). Frontal lobe inputs to the digit representations of the motor areas on the lateral surface of the hemisphere. J. Neurosci. 25, 1375–1386 10.1523/JNEUROSCI.3902-04.2005 15703391 PMC6726000

[B62] Duque J. Mazzocchio R. Stefan K. Hummel F. Olivier E. Cohen L. G. (2008). Memory formation in the motor cortex ipsilateral to a training hand. Cereb. Cortex 18, 1395–1406 10.1093/cercor/bhm173 17928331

[B63] Duque J. Murase N. Celnik P. Hummel F. Harris-Love M. Mazzocchio R. (2007). Intermanual differences in movement-related interhemispheric inhibition. J. Cogn. Neurosci. 19, 204–213 10.1162/jocn.2007.19.2.204 17280510

[B64] Farthing J. P. (2009). Cross-education of strength depends on limb dominance: implications for theory and application. Exerc. Sport Sci. Rev. 37, 179–187 19955867 10.1097/JES.0b013e3181b7e882

[B65] Farthing J. P. Borowsky R. Chilibeck P. D. Binsted G. Sarty G. E. (2007). Neuro-physiological adaptations associated with cross-education of strength. Brain Topogr. 20, 77–88 10.1007/s10548-007-0033-2 17932739

[B66] Farthing J. P. Krentz J. R. Magnus C. R. A. (2009). Strength training the free limb attenuates strength loss during unilateral immobilization. J. Appl. Physiol. 106, 830–836 10.1152/japplphysiol.91331.2008 19150859

[B67] Fenton A. A. Bures J. (1994). Interhippocampal transfer of place navigation monocularly acquired by rats during unilateral functional ablation of the dorsal hippocampus and visual cortex with lidocaine. Neuroscience 58, 481–491 10.1016/0306-4522(94)90074-4 8170534

[B68] Ferbert A. Priori A. Rothwell J. C. Day B. L. Colebatch J. G. Marsden C. D. (1992). Interhemispheric inhibition of the human motor cortex. J. Physiol. 453, 525–546 1464843 10.1113/jphysiol.1992.sp019243 PMC1175572

[B69] Fields R. D. (2008). White matter in learning, cognition and psychiatric disorders. Trends Neurosci. 31, 361–370 10.1016/j.tins.2008.04.001 18538868 PMC2486416

[B70] Fields R. D. (2011). Imaging learning: the search for a memory trace. Neuroscientist 17, 185–196 10.1177/1073858410383696 21403182 PMC3163891

[B72] Fling B. W. Benson B. L. Seidler R. D. (2011). Transcallosal sensorimotor fiber tract structure-function relationships. Hum. Brain Mapp. 34, 384–395 10.1002/hbm.21437 22042512 PMC3271163

[B71] Fling B. W. Seidler R. D. (2012). Fundamental differences in callosal structure, neurophysiologic function, and bimanual control in young and older adults. Cereb. Cortex 22, 2643–2652 10.1093/cercor/bhr349 22166764 PMC3464417

[B73] Floyer-Lea A. Matthews P. M. (2004). Changing brain networks for visuomotor control with increased movement automaticity. J. Neurophysiol. 92, 2405–2412 10.1152/jn.01092.2003 15381748

[B74] Francis S. Lin X. Aboushoushah S. White T. P. Phillips M. D. Bowtell R. (2009). fMRI analysis of active, passive and electrically stimulated ankle dorsiflexion. Neuroimage 44, 469–479 10.1016/j.neuroimage.2008.09.017 18950717

[B75] Friston K. J. Frith C. D. Passingham R. E. Liddle P. F. Frackowiak R. S. (1992). Motor practice and neurophysiological adaptation in the cerebellum: a positron tomography study. Proc. Biol. Sci. 248, 223–228 10.1098/rspb.1992.0065 1354360

[B76] Fu Q. G. Suarez J. I. Ebner T. J. (1993). Neuronal specification of direction and distance during reaching movements in the superior precentral premotor area and primary motor cortex of monkeys. J. Neurophysiol. 70, 2097–2116 8294972 10.1152/jn.1993.70.5.2097

[B77] Galea J. M. Vazquez A. Pasricha N. de Xivry J.-J. O. Celnik P. (2011). Dissociating the roles of the cerebellum and motor cortex during adaptive learning: the motor cortex retains what the cerebellum learns. Cereb. Cortex 21, 1761–1770 10.1093/cercor/bhq246 21139077 PMC3138512

[B78] Garry M. I. Loftus A. Summers J. J. (2005). Mirror, mirror on the wall: viewing a mirror reflection of unilateral hand movements facilitates ipsilateral M1 excitability. Exp. Brain Res. 163, 118–122 10.1007/s00221-005-2226-9 15754176

[B79] Gerloff C. Corwell B. Chen R. Hallett M. Cohen L. G. (1997). Stimulation over the human supplementary motor area interferes with the organization of future elements in complex motor sequences. Brain 120(Pt 9), 1587–1602 10.1093/brain/120.9.1587 9313642

[B80] Giovannelli F. Borgheresi A. Balestrieri F. Zaccara G. Viggiano M. P. Cincotta M. (2009). Modulation of interhemispheric inhibition by volitional motor activity: an ipsilateral silent period study. J. Physiol. 587, 5393–5410 10.1113/jphysiol.2009.175885 19770195 PMC2793872

[B81] Glover S. Miall R. C. Rushworth M. F. S. (2005). Parietal rTMS disrupts the initiation but not the execution of on-line adjustments to a perturbation of object size. J. Cogn. Neurosci. 17, 124–136 10.1162/0898929052880066 15701244

[B82] Grafton S. T. Hazeltine E. Ivry R. B. (2002). Motor sequence learning with the nondominant left hand. A PET functional imaging study. Exp. Brain Res. 146, 369–378 10.1007/s00221-002-1181-y 12232693

[B83] Grafton S. T. Woods R. P. Tyszka M. (1994). Functional imaging of procedural motor learning: relating cerebral blood flow with individual subject performance. Hum. Brain Mapp. 1, 221–234 10.1002/hbm.460010307 24578042

[B84] Griffin L. Cafarelli E. (2007). Transcranial magnetic stimulation during resistance training of the tibialis anterior muscle. J. Electromyogr. Kinesiol. 17, 446–452 10.1016/j.jelekin.2006.05.001 16891123

[B85] Halsband U. Lange R. K. (2006). Motor learning in man: a review of functional and clinical studies. J. Physiol. Paris 99, 414–424 10.1016/j.jphysparis.2006.03.007 16730432

[B86] Hanajima R. Ugawa Y. Machii K. Mochizuki H. Terao Y. Enomoto H. (2001). Interhemispheric facilitation of the hand motor area in humans. J. Physiol. 531, 849–859 10.1111/j.1469-7793.2001.0849h.x 11251064 PMC2278503

[B87] Hanakawa T. Parikh S. Bruno M. K. Hallett M. (2005). Finger and face representations in the ipsilateral precentral motor areas in humans. J. Neurophysiol. 93, 2950–2958 10.1152/jn.00784.2004 15625099 PMC1440886

[B88] Hardwick R. M. Rottschy C. Miall R. C. Eickhoff S. B. (2013). A quantitative meta-analysis and review of motor learning in the human brain. Neuroimage 67, 283–297 10.1016/j.neuroimage.2012.11.020 23194819 PMC3555187

[B89] He S. Q. Dum R. P. Strick P. L. (1993). Topographic organization of corticospinal projections from the frontal lobe: motor areas on the lateral surface of the hemisphere. J. Neurosci. 13, 952–980 7680069 10.1523/JNEUROSCI.13-03-00952.1993 PMC6576595

[B90] He S. Q. Dum R. P. Strick P. L. (1995). Topographic organization of corticospinal projections from the frontal lobe: motor areas on the medial surface of the hemisphere. J. Neurosci. 15, 3284–3306 7538558 10.1523/JNEUROSCI.15-05-03284.1995 PMC6578253

[B91] Hellebrandt F. A. (1951). Cross education; ipsilateral and contralateral effects of unimanual training. J. Appl. Physiol. 4, 136–144 14888623 10.1152/jappl.1951.4.2.136

[B92] Hicks R. E. Frank J. M. Kinsbourne M. (1982). The locus of bimanual skill transfer. J. Gen. Psychol. 107, 277–281 10.1080/00221309.1982.9709935 28150542

[B93] Hikosaka O. Nakahara H. Rand M. K. Sakai K. Lu X. Nakamura K. (1999). Parallel neural networks for learning sequential procedures. Trends Neurosci. 22, 464–471 10.1016/S0166-2236(99)01439-3 10481194

[B96] Hinder M. R. Schmidt M. W. Garry M. I. Carroll T. J. Summers J. J. (2011). Absence of cross-limb transfer of performance gains following ballistic motor practice in older adults. J. Appl. Physiol. 110, 166–175 10.1152/japplphysiol.00958.2010 21088207

[B94] Hinder M. R. Schmidt M. W. Garry M. I. Summers J. J. (2010a). The effect of ballistic thumb contractions on the excitability of the ipsilateral motor cortex. Exp. Brain Res. 201, 229–238 10.1007/s00221-009-2029-5 19826798

[B95] Hinder M. R. Schmidt M. W. Garry M. I. Summers J. J. (2010b). Unilateral contractions modulate interhemispheric inhibition most strongly and most adaptively in the homologous muscle of the contralateral limb. Exp. Brain Res. 205, 423–433 10.1007/s00221-010-2379-z 20686888

[B97] Hoffstaedter F. Grefkes C. Caspers S. Roski C. Fox P. T. Zilles K. (2012). Functional connectivity of the mid-cingulate cortex. Klin. Neurophysiol. 43, P128 10.1055/s-0032-1301678

[B98] Honda M. Deiber M. P. Ibáñez V. Pascual-Leone A. Zhuang P. Hallett M. (1998). Dynamic cortical involvement in implicit and explicit motor sequence learning. A PET study. Brain 121(Pt 11), 2159–2173 9827775 10.1093/brain/121.11.2159

[B99] Hopf H. C. Schlegel H. J. Lowitzsch K. (1974). Irradiation of voluntary activity to the contralateral side in movements of normal subjects and patients with central motor disturbances. Eur. Neurol. 12, 142–147 10.1159/000114613 4426322

[B100] Horenstein C. Lowe M. J. Koenig K. A. Phillips M. D. (2009). Comparison of unilateral and bilateral complex finger tapping-related activation in premotor and primary motor cortex. Hum. Brain Mapp. 30, 1397–1412 10.1002/hbm.20610 18537112 PMC6871138

[B101] Hortobagyi T. (2005). Cross education and the human central nervous system. IEEE Eng. Med. Biol. Mag. 24, 22–28 10.1109/MEMB.2005.1384096 15709532

[B102] Hortobagyi T. Dempsey L. Fraser D. Zheng D. Hamilton G. Lambert J. (2000). Changes in muscle strength, muscle fibre size and myofibrillar gene expression after immobilization and retraining in humans. J. Physiol. 524(Pt 1), 293–304 10.1111/j.1469-7793.2000.00293.x 10747199 PMC2269843

[B103] Hortobagyi T. Richardson S. P. Lomarev M. Shamim E. A. Meunier S. Russmann H. (2011). Interhemispheric plasticity in humans. Med. Sci. Sports Exerc. 43, 1188–1199 10.1249/MSS.0b013e31820a94b8 21200340 PMC4137570

[B104] Hortobagyi T. Taylor J. L. Petersen N. T. Russell G. Gandevia S. C. (2003). Changes in segmental and motor cortical output with contralateral muscle contractions and altered sensory inputs in humans. J. Neurophysiol. 90, 2451–2459 10.1152/jn.01001.2002 14534271

[B105] Houzel J. C. Milleret C. (1999). Visual inter-hemispheric processing: constraints and potentialities set by axonal morphology. J. Physiol. (Paris) 93, 271–284 10.1016/S0928-4257(00)80056-X 10574117

[B106] Imamizu H. Shimojo S. (1995). The locus of visual-motor learning at the task or manipulator level. Implications from intermanual transfer. J. Exp. Psychol. Hum. Percept. Perform. 21, 719–733 10.1037/0096-1523.21.4.719 7643045

[B107] Jantzen K. J. Steinberg F. L. Kelso J. A. S. (2002). Practice-dependent modulation of neural activity during human sensorimotor coordination: a functional Magnetic Resonance Imaging study. Neurosci. Lett. 332, 205–209 10.1016/S0304-3940(02)00956-4 12399015

[B108] Jenkins I. H. Brooks D. J. Nixon P. D. Frackowiak R. S. Passingham R. E. (1994). Motor sequence learning: a study with positron emission tomography. J. Neurosci. 14, 3775–3790 8207487 10.1523/JNEUROSCI.14-06-03775.1994 PMC6576955

[B109] Jenkins I. H. Passingham R. E. Brooks D. J. (1997). The effect of movement frequency on cerebral activation: a positron emission tomography study. J. Neurol. Sci. 151, 195–205 10.1016/S0022-510X(97)00145-7 9349676

[B110] Jenny A. B. (1979). Commissural projections of the cortical hand motor area in monkeys. J. Comp. Neurol. 188, 137–145 10.1002/cne.901880111 115906

[B111] Jensen J. L. Marstrand P. C. D. Nielsen J. B. (2005). Motor skill training and strength training are associated with different plastic changes in the central nervous system. J. Appl. Physiol. 99, 1558–1568 10.1152/japplphysiol.01408.2004 15890749

[B112] Jueptner M. Frith C. D. Brooks D. J. Frackowiak R. S. Passingham R. E. (1997a). Anatomy of motor learning. II. Subcortical structures and learning by trial and error. J. Neurophysiol. 77, 1325–1337 9084600 10.1152/jn.1997.77.3.1325

[B113] Jueptner M. Stephan K. M. Frith C. D. Brooks D. J. Frackowiak R. S. Passingham R. E. (1997b). Anatomy of motor learning. I. Frontal cortex and attention to action. J. Neurophysiol. 77, 1313–1324 9084599 10.1152/jn.1997.77.3.1313

[B114] Kang S. Y. Hallett M. Sohn Y. H. (2012). Synchronized finger exercise reduces surround inhibition. Clin. Neurophysiol. 123, 2227–2231 10.1016/j.clinph.2012.04.019 22608486

[B115] Kawashima R. Inoue K. Sato K. Fukuda H. (1997). Functional asymmetry of cortical motor control in left-handed subjects. Neuroreport 8, 1729–1732 10.1097/00001756-199705060-00032 9189922

[B116] Kermadi I. Liu Y. Rouiller E. M. (2000). Do bimanual motor actions involve the dorsal premotor (PMd), cingulate (CMA) and posterior parietal (PPC) cortices? Comparison with primary and supplementary motor cortical areas. Somatosens. Mot. Res. 17, 255–271 10.1080/08990220050117619 10994596

[B117] Kobayashi M. Hutchinson S. Schlaug G. (2003). Ipsilateral motor cortex activation on functional magnetic resonance imaging during unilateral hand movements is related to interhemispheric interactions. Neuroimage 20, 2259–2270 10.1016/S1053-8119(03)00220-9 14683727

[B118] Koch G. Cercignani M. Pecchioli C. Versace V. Oliveri M. Caltagirone C. (2010). *In vivo* definition of parieto-motor connections involved in planning of grasping movements. Neuroimage 51, 300–312 10.1016/j.neuroimage.2010.02.022 20156564

[B119] Koeneke S. Lutz K. Herwig U. Ziemann U. Jäncke L. (2006). Extensive training of elementary finger tapping movements changes the pattern of motor cortex excitability. Exp. Brain Res. 174, 199–209 10.1007/s00221-006-0440-8 16604315

[B120] Koeneke S. Lutz K. Wüstenberg T. Jäncke L. (2004). Bimanual versus unimanual coordination: what makes the difference? Neuroimage 22, 1336–1350 10.1016/j.neuroimage.2004.03.012 15219606

[B121] Koerte I. Heinen F. Fuchs T. Laubender R. P. Pomschar A. Stahl R. (2009). Anisotropy of callosal motor fibers in combination with transcranial magnetic stimulation in the course of motor development. Invest. Radiol. 44, 279–284 10.1097/RLI.0b013e31819e9362 19346962

[B122] Kogan A. B. Kuraev G. A. (1976). Organization of sensomotor cortical unit responses to stimulation of the symmetrical point of the opposite hemisphere. Neurosci. Behav. Physiol. 7, 174–177 10.1007/BF01320753

[B123] Koski L. Paus T. (2000). Functional connectivity of the anterior cingulate cortex within the human frontal lobe: a brain-mapping meta-analysis. Exp. Brain Res. 133, 55–65 10.1007/s002210000400 10933210

[B124] Kubota K. Hamada I. (1978). Visual tracking and neuron activity in the post-arcuate area in monkeys. J. Physiol. (Paris) 74, 297–312 102777

[B125] Kurata K. (1993). Premotor cortex of monkeys: set- and movement-related activity reflecting amplitude and direction of wrist movements. J. Neurophysiol. 69, 187–200 8433130 10.1152/jn.1993.69.1.187

[B126] Laszlo J. I. Baguley R. A. Bairstow P. J. (1970). Bilateral transfer in tapping skill in the absense of peripherial information. J. Mot. Behav. 2, 261–271 10.1080/00222895.1970.10734884 23941320

[B127] Latash M. L. (1999). Mirror writing: learning, transfer, and implications for internal inverse models. J. Mot. Behav. 31, 107–111 10.1080/00222899909600981 11177624

[B128] Lee D. Quessy S. (2003). Activity in the supplementary motor area related to learning and performance during a sequential visuomotor task. J. Neurophysiol. 89, 1039–1056 10.1152/jn.00638.2002 12574479

[B129] Lee M. Gandevia S. C. Carroll T. J. (2009). Unilateral strength training increases voluntary activation of the opposite untrained limb. Clin. Neurophysiol. 120, 802–808 10.1016/j.clinph.2009.01.002 19230754

[B130] Lee M. Hinder M. R. Gandevia S. C. Carroll T. J. (2010). The ipsilateral motor cortex contributes to cross-limb transfer of performance gains after ballistic motor practice. J. Physiol. 588, 201–212 10.1113/jphysiol.2009.183855 19917563 PMC2821559

[B131] Lenzi D. Conte A. Mainero C. Frasca V. Fubelli F. Totaro P. (2007). Effect of corpus callosum damage on ipsilateral motor activation in patients with multiple sclerosis: a functional and anatomical study. Hum. Brain Mapp. 28, 636–644 10.1002/hbm.20305 17080438 PMC6871400

[B132] Liang N. Takahashi M. Ni Z. Yahagi S. Funase K. Kato T. (2007). Effects of intermanual transfer induced by repetitive precision grip on input–output properties of untrained contralateral limb muscles. Exp. Brain Res. 182, 459–467 10.1007/s00221-007-1004-2 17562034

[B133] Liepert J. Classen J. Cohen L. G. Hallett M. (1998). Task-dependent changes of intracortical inhibition. Exp. Brain Res. 118, 421–426 10.1007/s002210050296 9497149

[B134] Liepert J. Dettmers C. Terborg C. Weiller C. (2001). Inhibition of ipsilateral motor cortex during phasic generation of low force. Clin. Neurophysiol. 112, 114–121 10.1016/S1388-2457(00)00503-4 11137668

[B135] Liu J. Morel A. Wannier T. Rouiller E. M. (2002). Origins of callosal projections to the supplementary motor area (SMA): a direct comparison between pre-SMA and SMA-proper in macaque monkeys. J. Comp. Neurol. 443, 71–85 10.1002/cne.10087 11793348

[B136] Magnus C. R. A. Barss T. S. Lanovaz J. L. Farthing J. P. (2010). Effects of cross-education on the muscle after a period of unilateral limb immobilization using a shoulder sling and swathe. J. Appl. Physiol. 109, 1887–1894 10.1152/japplphysiol.00597.2010 20966196

[B137] Makris N. Kennedy D. N. McInerney S. Sorensen A. G. Wang R. Caviness V. S. (2005). Segmentation of subcomponents within the superior longitudinal fascicle in humans: a quantitative, *in vivo*, DT-MRI study. Cereb. Cortex 15, 854–869 10.1093/cercor/bhh186 15590909

[B138] Manto M. Godaux E. Jacquy J. (1994). Cerebellar hypermetria is larger when the inertial load is artificially increased. Ann. Neurol. 35, 45–52 10.1002/ana.410350108 8285591

[B139] Martin T. A. Keating J. G. Goodkin H. P. Bastian A. J. (1996). Throwing while looking through prisms II. Specificity and storage of multiple gaze—throw calibrations. Brain 119, 1199–1211 10.1093/brain/119.4.1199 8813283

[B140] Mayston M. J. Harrison L. M. Stephens J. A. (1999). A neurophysiological study of mirror movements in adults and children. Ann. Neurol. 45, 583–594 10319880 10.1002/1531-8249(199905)45:5<583::aid-ana6>3.0.co;2-w

[B141] McCombe Waller S. Forrester L. Villagra F. Whitall J. (2008). Intracortical inhibition and facilitation with unilateral dominant, unilateral nondominant and bilateral movement tasks in left- and right-handed adults. J. Neurol. Sci. 269, 96–104 10.1016/j.jns.2007.12.033 18336839 PMC2910578

[B143] Meyer B. U. Röricht S. Gräfin von Einsiedel H. Kruggel F. Weindl A. (1995). Inhibitory and excitatory interhemispheric transfers between motor cortical areas in normal humans and patients with abnormalities of the corpus callosum. Brain 118(Pt 2), 429–440 10.1093/brain/118.2.429 7735884

[B142] Meyer B. U. Röricht S. Woiciechowsky C. (1998). Topography of fibers in the human corpus callosum mediating interhemispheric inhibition between the motor cortices. Ann. Neurol. 43, 360–369 10.1002/ana.410430314 9506553

[B145] Miall R. C. Christensen L. O. D. Cain O. Stanley J. (2007). Disruption of state estimation in the human lateral cerebellum. PLoS Biol. 5:e316 10.1371/journal.pbio.0050316 18044990 PMC2229864

[B144] Miall R. C. King D. (2008). State estimation in the cerebellum. Cerebellum 7, 572–576 10.1007/s12311-008-0072-6 18855092 PMC6010151

[B146] Milner A. D. Goodale M. A. (1993). Visual pathways to perception and action. Prog. Brain Res. 95, 317–337 10.1016/S0079-6123(08)60379-9 8493342

[B147] Mountcastle V. B. Lynch J. C. Georgopoulos A. Sakata H. Acuna C. (1975). Posterior parietal association cortex of the monkey: command functions for operations within extrapersonal space. J. Neurophysiol. 38, 871–908 808592 10.1152/jn.1975.38.4.871

[B148] Müller R. A. Kleinhans N. Pierce K. Kemmotsu N. Courchesne E. (2002). Functional MRI of motor sequence acquisition: effects of learning stage and performance. Brain Res. Cogn. Brain Res. 14, 277–293 10.1016/S0926-6410(02)00131-3 12067701

[B149] Münchau A. Bloem B. R. Irlbacher K. Trimble M. R. Rothwell J. C. (2002). Functional connectivity of human premotor and motor cortex explored with repetitive transcranial magnetic stimulation. J. Neurosci. 22, 554–561 11784802 10.1523/JNEUROSCI.22-02-00554.2002 PMC6758651

[B150] Nadel N. Buresova O. (1968). Monocular input and interhemispheric transfer in the reversible split-brain. Nature 220, 914–915 10.1038/220914a0 5722141

[B151] Nissen M. J. Bullemer P. (1987). Attentional requirements of learning: evidence from performance measures. Cogn. Psychol. 19, 1–32 10.1016/0010-0285(87)90002-8

[B152] Obayashi S. (2004). Possible mechanism for transfer of motor skill learning: implication of the cerebellum. Cerebellum 3, 204–211 10.1080/14734220410018977 15686098

[B153] Ohtsuki T. (1983). Decrease in human voluntary isometric arm strength induced by simultaneous bilateral exertion. Behav. Brain Res. 7, 165–178 10.1016/0166-4328(83)90190-0 6830650

[B154] Orban de Xivry J.-J. Criscimagna-Hemminger S. E. Shadmehr R. (2011). Contributions of the motor cortex to adaptive control of reaching depend on the perturbation schedule. Cereb. Cortex 21, 1475–1484 10.1093/cercor/bhq192 21131448 PMC3116732

[B155] Ortu E. Ruge D. Deriu F. Rothwell J. C. (2009). Theta Burst Stimulation over the human primary motor cortex modulates neural processes involved in movement preparation. Clin. Neurophysiol. 120, 1195–1203 10.1016/j.clinph.2009.04.001 19410505

[B156] Padoa-Schioppa C. Li C. S. R. Bizzi E. (2002). Neuronal correlates of kinematics-to-dynamics transformation in the supplementary motor area. Neuron 36, 751–765 10.1016/S0896-6273(02)01028-0 12441062

[B157] Pal P. K. (2005). Effect of low-frequency repetitive transcranial magnetic stimulation on interhemispheric inhibition. J. Neurophysiol. 94, 1668–1675 10.1152/jn.01306.2004 15872061

[B158] Parlow S. E. Dewey D. (1991). The temporal locus of transfer of training between hands: an interference study. Behav. Brain Res. 46, 1–8 10.1016/S0166-4328(05)80091-9 1786110

[B159] Parlow S. E. Kinsbourne M. (1989). Asymmetrical transfer of training between hands: implications for interhemispheric communication in normal brain. Brain Cogn. 11, 98–113 10.1016/0278-2626(89)90008-0 2789820

[B160] Pearce A. J. Hendy A. Bowen W. A. Kidgell D. J. (2012). Corticospinal adaptations and strength maintenance in the immobilized arm following 3 weeks unilateral strength training. Scand. J. Med. Sci. Sports. 10.1111/j.1600-0838.2012.01453.x 22429184

[B161] Peltier J. Roussel M. Gerard Y. Lassonde M. Deramond H. Gars D. L. (2012). Functional consequences of a section of the anterior part of the body of the corpus callosum: evidence from an interhemispheric transcallosal approach. J. Neurol. 259, 1860–1867 10.1007/s00415-012-6421-x 22289969

[B161a] Perez M. A. (2012). The functional role of interhemispheric interactions in human motor control, in Cortical Connectivity: Brain Stimulation for Assessing and Modulating Cortical Connectivity and Function, eds Chen R. Rothwell J. C. (Berlin; Heidelberg: Springer), 165–181

[B162] Perez M. A. Cohen L. G. (2008). Mechanisms underlying functional changes in the primary motor cortex ipsilateral to an active hand. J. Neurosci. 28, 5631–5640 10.1523/JNEUROSCI.0093-08.2008 18509024 PMC2440822

[B163] Perez M. A. Tanaka S. Wise S. P. Sadato N. Tanabe H. C. Willingham D. T. (2007a). Neural substrates of intermanual transfer of a newly acquired motor skill. Curr. Biol. 17, 1896–1902 10.1016/j.cub.2007.09.058 17964167

[B164] Perez M. A. Wise S. P. Willingham D. T. Cohen L. G. (2007b). Neurophysiological mechanisms involved in transfer of procedural knowledge. J. Neurosci. 27, 1045–1053 10.1523/JNEUROSCI.4128-06.2007 17267558 PMC6673204

[B165] Perez M. A. Tanaka S. Wise S. P. Willingham D. T. Cohen L. G. (2008). Time-specific contribution of the supplementary motor area to intermanual transfer of procedural knowledge. J. Neurosci. 28, 9664–9669 10.1523/JNEUROSCI.3416-08.2008 18815252 PMC2569889

[B166] Poh E. Riek S. Carroll T. J. (2013). Ipsilateral corticospinal responses to ballistic training are similar for various intensities and timings of TMS. Acta Physiol. (Oxf.) 207, 385–396 10.1111/apha.12032 23082845

[B167] Richardson A. G. Overduin S. A. Valero-Cabre A. Padoa-Schioppa C. Pascual-Leone A. Bizzi E. (2006). Disruption of primary motor cortex before learning impairs memory of movement dynamics. J. Neurosci. 26, 12466–12470 10.1523/JNEUROSCI.1139-06.2006 17135408 PMC6674906

[B168] Riek S. Hinder M. R. Carson R. G. (2012). Primary motor cortex involvement in initial learning during visuomotor adaptation. Neuropsychologia 50, 2515–2523 10.1016/j.neuropsychologia.2012.06.024 22781812

[B169] Rizzolatti G. Luppino G. Matelli M. (1998). The organization of the cortical motor system: new concepts. Electroencephalogr. Clin. Neurophysiol. 106, 283–296 10.1016/S0013-4694(98)00022-4 9741757

[B170] Robertson E. M. (2007). The serial reaction time task: implicit motor skill learning? J. Neurosci. 27, 10073–10075 10.1523/JNEUROSCI.2747-07.2007 17881512 PMC6672677

[B171] Rosenkranz K. Kacar A. Rothwell J. C. (2007). Differential modulation of motor cortical plasticity and excitability in early and late phases of human motor learning. J. Neurosci. 27, 12058–12066 10.1523/JNEUROSCI.2663-07.2007 17978047 PMC6673358

[B172] Rouiller E. M. Babalian A. Kazennikov O. Moret V. Yu X. H. Wiesendanger M. (1994). Transcallosal connections of the distal forelimb representations of the primary and supplementary motor cortical areas in macaque monkeys. Exp. Brain Res. 102, 227–243 10.1007/BF00227511 7705502

[B173] Rouiller E. M. Yu X. H. Moret V. Tempini A. Wiesendanger M. Liang F. (1998). Dexterity in adult monkeys following early lesion of the motor cortical hand area: the role of cortex adjacent to the lesion. Eur. J. Neurosci. 10, 729–740 10.1046/j.1460-9568.1998.00075.x 9749734

[B174] Sadato N. Ibáñez V. Deiber M. P. Campbell G. Leonardo M. Hallett M. (1996). Frequency-dependent changes of regional cerebral blood flow during finger movements. J. Cereb. Blood Flow Metab. 16, 23–33 8530552 10.1097/00004647-199601000-00003

[B175] Sainburg R. L. Wang J. (2002). Interlimb transfer of visuomotor rotations: independence of direction and final position information. Exp. Brain Res. 145, 437–447 10.1007/s00221-002-1140-7 12172655 PMC10704413

[B176] Sakai K. Hikosaka O. Miyauchi S. Takino R. Sasaki Y. Pütz B. (1998). Transition of brain activation from frontal to parietal areas in visuomotor sequence learning. J. Neurosci. 18, 1827–1840 9465007 10.1523/JNEUROSCI.18-05-01827.1998 PMC6792634

[B177] Salerno A. Georgesco M. (1996). Interhemispheric facilitation and inhibition studied in man with double magnetic stimulation. Electroencephalogr. Clin. Neurophysiol. 101, 395–403 8913192

[B178] Sanes J. N. Donoghue J. P. (2000). Plasticity and primary motor cortex. Annu. Rev. Neurosci. 23, 393–415 10.1146/annurev.neuro.23.1.393 10845069

[B179] Schlaug G. Knorr U. Seitz R. (1994). Inter-subject variability of cerebral activations in acquiring a motor skill: a study with positron emission tomography. Exp. Brain Res. 98, 523–534 10.1007/BF00233989 8056072

[B180] Schubotz R. I. von Cramon D. Y. (2002). A blueprint for target motion: fMRI reveals perceived sequential complexity to modulate premotor cortex. Neuroimage 16, 920–935 10.1006/nimg.2002.1183 12202080

[B181] Schubotz R. I. von Cramon D. Y. (2003). Functional-anatomical concepts of human premotor cortex: evidence from fMRI and PET studies. Neuroimage 20 Suppl. 1, S120–S131 10.1016/j.neuroimage.2003.09.014 14597305

[B182] Scripture E. W. Smith T. L. Brown E. M. (1894). On the education of muscular control and power. Stud. Yale Psychol. Lab. 2, 114–119

[B183] Seidler R. D. Bernard J. A. Burutolu T. B. Fling B. W. Gordon M. T. Gwin J. T. (2010). Motor control and aging: links to age-related brain structural, functional, and biochemical effects. Neurosci. Biobehav. Rev. 34, 721–733 10.1016/j.neubiorev.2009.10.005 19850077 PMC2838968

[B184] Seidler R. D. Purushotham A. Kim S. G. Uğurbil K. Willingham D. Ashe J. (2005). Neural correlates of encoding and expression in implicit sequence learning. Exp. Brain Res. 165, 114–124 10.1007/s00221-005-2284-z 15965762

[B186] Seitz R. J. Canavan A. G. Yágüez L. Herzog H. Tellmann L. Knorr U. (1994). Successive roles of the cerebellum and premotor cortices in trajectorial learning. Neuroreport 5, 2541–2544 10.1097/00001756-199412000-00034 7696599

[B185] Seitz R. J. Roland P. E. (1992). Learning of sequential finger movements in man: a combined kinematic and positron emission tomography (PET) study. Eur. J. Neurosci. 4, 154–165 10.1111/j.1460-9568.1992.tb00862.x 12106378

[B187] Shemmell J. Riek S. Tresilian J. R. Carson R. G. (2007). The role of the primary motor cortex during skill acquisition on a two-degrees-of-freedom movement task. J. Mot. Behav. 39, 29–39 10.3200/JMBR.39.1.29-39 17251169

[B188] Shima K. Tanji J. (1998). Both supplementary and presupplementary motor areas are crucial for the temporal organization of multiple movements. J. Neurophysiol. 80, 3247–3260 9862919 10.1152/jn.1998.80.6.3247

[B189] Shima K. Tanji J. (2000). Neuronal activity in the supplementary and presupplementary motor areas for temporal organization of multiple movements. J. Neurophysiol. 84, 2148–2160 11024102 10.1152/jn.2000.84.4.2148

[B190] Siebner H. R. Hartwigsen G. Kassuba T. Rothwell J. C. (2009). How does transcranial magnetic stimulation modify neuronal activity in the brain? Implications for studies of cognition. Cortex 45, 1035–1042 10.1016/j.cortex.2009.02.007 19371866 PMC2997692

[B191] Singh L. N. Higano S. Takahashi S. Abe Y. Sakamoto M. Kurihara N. (1998a). Functional MR imaging of cortical activation of the cerebral hemispheres during motor tasks. AJNR Am. J. Neuroradiol. 19, 275–280 9504477 PMC8338169

[B192] Singh L. N. Higano S. Takahashi S. Kurihara N. Furuta S. Tamura H. (1998b). Comparison of ipsilateral activation between right and left handers: a functional MR imaging study. Neuroreport 9, 1861–1866 10.1097/00001756-199806010-00036 9665616

[B193] Sosnik R. (2010). Practice makes bimanual interference imperfect–On the way to the generation of bimanual motion primitives. Cortex 46, 264–267 10.1016/j.cortex.2009.02.008 19321164

[B194] Stanæák A. Cohen E. R. Seidler R. D. Duong T. Q. Kim S.-G. (2003). The size of corpus callosum correlates with functional activation of medial motor cortical areas in bimanual and unimanual movements. Cereb. Cortex 13, 475–485 10.1093/cercor/13.5.475 12679294

[B195] Sullivan E. V. Rohlfing T. Pfefferbaum A. (2010). Quantitative fiber tracking of lateral and interhemispheric white matter systems in normal aging: relations to timed performance. Neurobiol. Aging 31, 464–481 10.1016/j.neurobiolaging.2008.04.007 18495300 PMC2815144

[B196] Szameitat A. J. Shen S. Conforto A. Sterr A. (2012). Cortical activation during executed, imagined, observed, and passive wrist movements in healthy volunteers and stroke patients. Neuroimage 62, 266–280 10.1016/j.neuroimage.2012.05.009 22584231

[B197] Talelli P. Ewas A. Waddingham W. Rothwell J. C. (2008a). Neural correlates of age-related changes in cortical neurophysiology. Neuroimage 40, 1772–1781 10.1016/j.neuroimage.2008.01.039 18329904 PMC3715371

[B198] Talelli P. Waddingham W. Ewas A. Rothwell J. C. Ward N. S. (2008b). The effect of age on task-related modulation of interhemispheric balance. Exp. Brain Res. 186, 59–66 10.1007/s00221-007-1205-8 18040671 PMC2257995

[B199] Tanji J. Shima K. (1994). Role for supplementary motor area cells in planning several movements ahead. Nature 371, 413–416 10.1038/371413a0 8090219

[B200] Taylor H. G. Heilman K. M. (1980). Left-hemisphere motor dominance in righthanders. Cortex 16, 587–603 10.1016/S0010-9452(80)80006-2 7226856

[B201] Todor J. I. Lazarus J. A. (1986). Exertion level and the intensity of associated movements. Dev. Med. Child Neurol. 28, 205–212 10.1111/j.1469-8749.1986.tb03856.x 3709990

[B202] Touge T. Gerschlager W. Brown P. Rothwell J. C. (2001). Are the after-effects of low-frequency rTMS on motor cortex excitability due to changes in the efficacy of cortical synapses? Clin. Neurophysiol. 112, 2138–2145 10.1016/S1388-2457(01)00651-4 11682353

[B203] Tseng Y. W. Diedrichsen J. Krakauer J. W. Shadmehr R. Bastian A. J. (2007). Sensory prediction errors drive cerebellum-dependent adaptation of reaching. J. Neurophysiol. 98, 54–62 10.1152/jn.00266.2007 17507504

[B204] Tsuboi F. Nishimura Y. Yoshino-Saito K. Isa T. (2010). Neuronal mechanism of mirror movements caused by dysfunction of the motor cortex. Eur. J. Neurosci. 32, 1397–1406 10.1111/j.1460-9568.2010.07395.x 20846329

[B205] Turner R. S. Grafton S. T. Votaw J. R. Delong M. R. Hoffman J. M. (1998). Motor subcircuits mediating the control of movement velocity: a PET study. J. Neurophysiol. 80, 2176–2176 9772269 10.1152/jn.1998.80.4.2162

[B206] Uehara K. Morishita T. Kubota S. Funase K. (2013). Neural mechanisms underlying the changes in ipsilateral primary motor cortex excitability during unilateral rhythmic muscle contraction. Behav. Brain Res. 240, 33–45 10.1016/j.bbr.2012.10.053 23174210

[B207] Ugawa Y. Hanajima R. Kanazawa I. (1993). Interhemispheric facilitation of the hand area of the human motor cortex. Neurosci. Lett. 160, 153–155 10.1016/0304-3940(93)90401-6 8247346

[B208] van Duinen H. Renken R. Maurits N. M. Zijdewind I. (2008). Relation between muscle and brain activity during isometric contractions of the first dorsal interosseus muscle. Hum. Brain Mapp. 29, 281–299 10.1002/hbm.20388 17394210 PMC6870705

[B209] Vercauteren K. Pleysier T. Van Belle L. Swinnen S. P. Wenderoth N. (2008). Unimanual muscle activation increases interhemispheric inhibition from the active to the resting hemisphere. Neurosci. Lett. 445, 209–213 10.1016/j.neulet.2008.09.013 18793696

[B211] Verstynen T. Diedrichsen J. Albert N. Aparicio P. Ivry R. B. (2005). Ipsilateral motor cortex activity during unimanual hand movements relates to task complexity. J. Neurophysiol. 93, 1209–1222 10.1152/jn.00720.2004 15525809

[B210] Verstynen T. Ivry R. B. (2011). Network dynamics mediating ipsilateral motor cortex activity during unimanual actions. J. Cogn. Neurosci. 23, 2468–2480 10.1162/jocn.2011.21612 21268666

[B212] Vollmann H. Conde V. Sewerin S. Taubert M. Sehm B. (2012). Anodal transcranial direct current stimulation (tDCS) over supplementary motor area (SMA) but not pre-SMA promotes short-term visuomotor learning. Brain Stimul. 6, 101–107 10.1016/j.brs.2012.03.018 22659022

[B213] Wahl M. Lauterbach-Soon B. Hattingen E. Jung P. Singer O. Volz S. (2007). Human motor corpus callosum: topography, somatotopy, and link between microstructure and function. J. Neurosci. 27, 12132–12138 10.1523/JNEUROSCI.2320-07.2007 17989279 PMC6673264

[B214] Waldvogel D. van Gelderen P. Muellbacher W. Ziemann U. Immisch I. Hallett M. (2000). The relative metabolic demand of inhibition and excitation. Nature 406, 995–998 10.1038/35023171 10984053

[B215] Walters C. E. (1955). The effect of overload on bilateral transfer of a motor skill. Phys. Ther. Rev. 35, 567–569 13266520 10.1093/ptj/35.10.567

[B216] Wassermann E. M. Fuhr P. Cohen L. G. Hallett M. (1991). Effects of transcranial magnetic stimulation on ipsilateral muscles. Neurology 41, 1795–1799 10.1212/WNL.41.11.1795 1944911

[B216a] Welch J. C. (1898). On the measurement of mental activity through muscular activity and the determination of a constant of attention. Am. J. Physiol. 1, 283–306

[B217] Werhahn K. J. Kunesch E. Noachtar S. Benecke R. Classen J. (1999). Differential effects on motorcortical inhibition induced by blockade of GABA uptake in humans. J. Physiol. 517(Pt 2), 591–597 10.1111/j.1469-7793.1999.0591t.x 10332104 PMC2269337

[B218] Winterer G. Adams C. M. Jones D. W. Knutson B. (2002). Volition to action–an event-related fMRI study. Neuroimage 17, 851–858 10.1006/nimg.2002.1232 12377159

[B219] Wise S. P. Boussaoud D. Johnson P. B. Caminiti R. (1997). Premotor and parietal cortex: corticocortical connectivity and combinatorial computations. Annu. Rev. Neurosci. 20, 25–42 10.1146/annurev.neuro.20.1.25 9056706

[B220] Yu N. Estévez N. Hepp-Reymond M. C. Kollias S. S. Riener R. (2011). fMRI assessment of upper extremity related brain activation with an MRI-compatible manipulandum. Int. J. Comput. Assist. Radiol. Surg. 6, 447–455 10.1007/s11548-010-0525-5 20697829

[B221] Zhou S. (2000). Chronic neural adaptations to unilateral exercise: mechanisms of cross education. Exerc. Sport Sci. Rev. 28, 177–184 11064852

